# The *Edwardsiella* T3SS effector EseJ enhances NF-κB signaling by stabilizing p65 and promoting TAK1 phosphorylation

**DOI:** 10.1371/journal.ppat.1014260

**Published:** 2026-09-15

**Authors:** Tian Tian He, Pu Yu Tang, Qian Ru Zhao, Jie Zhang, Pin Nie, Hong Bing Yu, Hai Xia Xie

**Affiliations:** 1 Institute of Hydrobiology, Chinese Academy of Sciences, Wuhan, China; 2 State Key Laboratory of Breeding Biotechnology and Sustainable Aquaculture, Institute of Hydrobiology, Chinese Academy of Sciences, Wuhan, China; 3 Universityof Chinese Academy of Sciences, Beijing, China; 4 Department of Microbiology, Molecular Genetics and Immunology, University of Kansas Medical Centre, Kansas, Missouri, United States of America; Johns Hopkins University Bloomberg School of Public Health, UNITED STATES OF AMERICA

## Abstract

*Edwardsiella piscicida* PPD130/91 is an important pathogen that infects both fish and humans. Upon infection, *E. piscicida* induces strong inflammatory responses through its type III secretion system (T3SS). The underlying mechanisms remain to be defined. Here, we demonstrate that *E. piscicida* T3SS effector EseJ activates the NF-κB pathway in macrophages, leading to increased transcription and secretion of multiple pro-inflammatory cytokines (e.g., IL-1β and IL-6). EseJ orchestrates NF-κB signaling by interfering with two ubiquitin-dependent mechanisms that are mediated by separate functional domains of EseJ. EseJ binds to the NF-κB subunit p65 via its N-terminal region of amino acids 1–242, preventing the K48-linked polyubiquitination of p65 and its subsequent degradation by the proteasome. This maintains elevated levels of total and phosphorylated p65 (p-p65), facilitating p65 nuclear translocation and enhancing its transcriptional activity. EseJ also interacts with TAK1 and TRAF6 (both are upstream of the NF-κB signaling pathway) via its central domain (aa 243–532), thereby promoting the formation of a stable TRAF6-TAK1 complex. This interaction increases the TRAF6-dependent K27-linked polyubiquitination of TAK1, driving TAK1 phosphorylation and the subsequent activation of the IKK-IκB-NF-κB cascade. Inhibiting TAK1 abrogates this signaling, confirming the essential role of TAK1 in EseJ-induced NF-κB activation. *In vivo* studies using zebrafish larvae also demonstrated that EseJ is required for *E. piscicida* to induce the production of pro-inflammatory cytokines. Together, we provide evidence that EseJ targets both TAK1 and p65 to promote NF-κB-dependent inflammatory responses. Given the similarities among EseJ homologues in other bacteria, our finding may reveal a conserved effector-mediated immune signaling across diverse Gram-negative pathogens.

## Author summary

Bacterial pathogens often use specialized molecular syringes, known as type III secretion systems, to inject effector proteins directly into host cells and manipulate immune defenses. *Edwardsiella piscicida* is a significant pathogen affecting aquaculture and human health, known for triggering inflammation, though the precise bacterial factors driving this response have remained unclear. In this study, we identify the secreted bacterial effector EseJ as a central driver of host inflammatory signaling. We demonstrate that EseJ functions through a dual-action mechanism, using two distinct structural regions to hijack host ubiquitin pathways and overactivate the immune regulator NF-κB. Specifically, EseJ stabilizes the key signaling protein TAK1 while simultaneously protecting the transcription factor p65 from degradation, leading to sustained production of pro-inflammatory cytokines. Disruption of EseJ reduces inflammation during infection in zebrafish models, confirming its critical role *in vivo*. Because EseJ is highly conserved across *Edwardsiella* species, our findings reveal an underappreciated strategy used by these pathogens to exacerbate host inflammation, offering new insights into bacterial virulence and potential therapeutic targets for combating infection.

## Introduction

*Edwardsiella piscicida* (formerly *Edwardsiella tarda* PPD130/91) is a Gram-negative, facultative intracellular pathogen that causes haemorrhagic septicaemia in various economically important fish species [1,2]. Beyond its impact on aquaculture, it is increasingly recognized as an emerging cause of human infection [3,4]. Central to the pathogenesis of *E. piscicida* is its type III secretion system (T3SS), a molecular syringe used to inject effector proteins directly into the host cell cytoplasm. This specialized apparatus is indispensable for subverting host innate immunity, facilitating intracellular survival, and establishing a replicative niche within host macrophages [5].

In *E. piscicida*, the precise assembly of the T3SS needle complex (EscE-EsaG-EsaH apparatus) and the subsequent secretion of the needle protein EsaG are essential prerequisites for effector translocation [6]. Once delivered into the host cytosol, these effectors systematically subvert host cellular processes, including the modulation of programmed cell death and immune signaling, to facilitate bacterial colonization and systemic dissemination [7–9]. A suite of T3SS effectors, including EseG, EseJ, EseH, EseK, Trx2, and YfiD, have been identified as important for *E. piscicida* virulence [10–15]. Among these, EseJ stands out as a unique bifunctional protein, acting as both an effector and a transcriptional regulator [11,16]. As an effector, EseJ promotes *in vivo* replication by suppressing the production of reactive oxygen species (ROS) and the fusion of lysosomes with *E. piscicida*-containing vacuoles [11,17]. Conversely, as a regulator, EseJ suppresses the transcription of the type 1 fimbrial operon, limiting bacterial adhesion and invasion into epithelial cells [16]. Despite these insights into its role in vacuolar trafficking and fimbrial expression, the extent to which EseJ modulates host innate immune signaling, such as nuclear factor kappa-B (NF-κB) activation, remains a critical gap in our understanding of *E. piscicida* pathogenesis.

The NF-κB transcription factor family, comprising p50 (NF-κB1), p52 (NF-κB2), RelA (p65), RelB, and c-Rel, is a master regulator of the host inflammatory response [18]. In quiescent cells, the p65/p50 heterodimer is sequestered in the cytoplasm through association with the inhibitor nuclear factor kappa-B kinase (IκB) [19]. Upon sensing stress signals or pathogen-associated molecular patterns (PAMPs), the IκB kinase (IKK) complex phosphorylates IκBα, triggering its proteasomal degradation. This liberation allows the p65/p50 complex to translocate into the nucleus and drive the transcription of key pro-inflammatory cytokines, such as IL-1β, IL-6 and IL-18 [20]. The activity of p65 is strictly governed by coordinated post-translational modifications [21]. Phosphorylation at Ser276 and Ser311, mediated by kinases such as PKA or MSK1, is essential for stabilizing the p65 transcriptional complex [22,23]. Furthermore, ubiquitination exerts a dual regulatory role: while K48-linked polyubiquitination targets p65 for proteasomal turnover to prevent excessive inflammation [24,25], K63-linked ubiquitination (e.g., via TRIM21) enhances its nuclear trafficking and transcriptional activity [26]. Given this complex regulatory role, many intracellular pathogens have evolved effectors to specifically manipulate these modifications to tune the host immune response.

Transforming growth factor-β-activated kinase 1 (TAK1) is a critical upstream regulator of the NF-κB pathway. It coordinates the phosphorylation of the IKK complex, thereby facilitating the nuclear translocation of p65 [27]. The catalytic activity of TAK1 is strictly regulated by post-translational modifications, primarily polyubiquitination and autophosphorylation. Specifically, TRAF6 mediates both K63- and K27-linked ubiquitination of TAK1 to initiate signaling [28,29]. K63-linked polyubiquitination induces conformational changes that trigger TAK1 autophosphorylation at residues Thr187, Thr178, Thr184, and Ser192 [30,31]. K27-linked polyubiquitination is essential for the recruitment of the adapter proteins TAB2 and TAB3. The formation of this heterotrimeric TAK1-TAB complex is a prerequisite for the subsequent phosphorylation of IKKβ, bridging upstream stimuli to the canonical NF-κB cascade [29].

While the NF-κB signaling pathway is indispensable for pathogen detection and clearance, many Gram-negative bacteria deploy T3SS effectors to subvert this axis and facilitate *in vivo* persistence [32–34]. For example, the enterohemorrhagic *Escherichia coli* (EHEC) effector NleH1 inhibits the phosphorylation and nuclear translocation of the co-activator RPS3, thereby dampening the transcription of pro-inflammatory genes such as *il-8* and *tnf-α* [35,36]. Similarly, the *Salmonella* effector SpvD binds the nuclear export protein XPO2, disrupting the importin-α (KPNA1/3) shuttle system to prevent the nuclear entry of p65 [33]. In striking contrast to these inhibitory effects, early evidence suggests that *E. piscicida* stimulates the NF-κB pathway. Compared to T3SS-deficient mutants, wild-type *E. piscicida* infection significantly upregulates NF-κB target genes, including pro-inflammatory cytokines and anti-apoptotic factors in macrophages [37]. Despite the significance of this observation for *Edwardsiella* pathogenesis, the specific T3SS effector(s) responsible for this activation and the molecular mechanisms by which they engage the host signaling machinery have yet to be defined.

In this study, we uncover that the *E. piscicida* T3SS effector EseJ is a potent activator of the NF-κB signaling, employing a unique bimodal strategy to augment inflammatory responses. We demonstrate that EseJ functions as a molecular scaffold, using distinct functional domains to simultaneously stabilize the core transcription factor and accelerate its upstream activation. The N-terminal domain (aa 1–242) of EseJ interacts with p65, preventing it from K48-linked polyubiquitination and subsequent proteasomal turnover. The central domain (aa 243–532) of EseJ binds to TAK1 and TRAF6, promoting TRAF6-mediated K27-linked polyubiquitination and the autophosphorylation of TAK1. Consistent with this dual-action mechanism, we show that EseJ is indispensable for the inflammatory responses observed across *in vitro*, *ex vivo*, and *in vivo* zebrafish infection models.

## Results

### *E. piscicida*-mediated NF-κB activation in J774A.1 macrophages requires EseJ

While *E. piscicida* uses its T3SS to induce NF-κB target genes [37], the T3SS effector(s) required for this induction remains unclear. Our previous finding that EseJ promotes the secretion of the pro-inflammatory cytokine interleukin-1β (IL-1β) [38], a cytokine downstream of NF-κB signaling, led us to hypothesize that EseJ is the specific effector required for NF-κB activation during infection. To test this hypothesis, we infected J774A.1 macrophages with *E. piscicida* Δ*fimH*, Δ*eseJ*Δ*fimH*, Δ*fimA*, and Δ*eseJ*Δ*fimA*, all of which adhere to host cells at similar levels [16]. We specifically excluded wild-type and Δ*eseJ* strains to avoid confounding effects associated with EseJ-mediated suppression of type 1 fimbriae [16], ensuring that any observed differences in host response were due to EseJ effector function rather than differential bacterial adherence.

**Fig 1:**
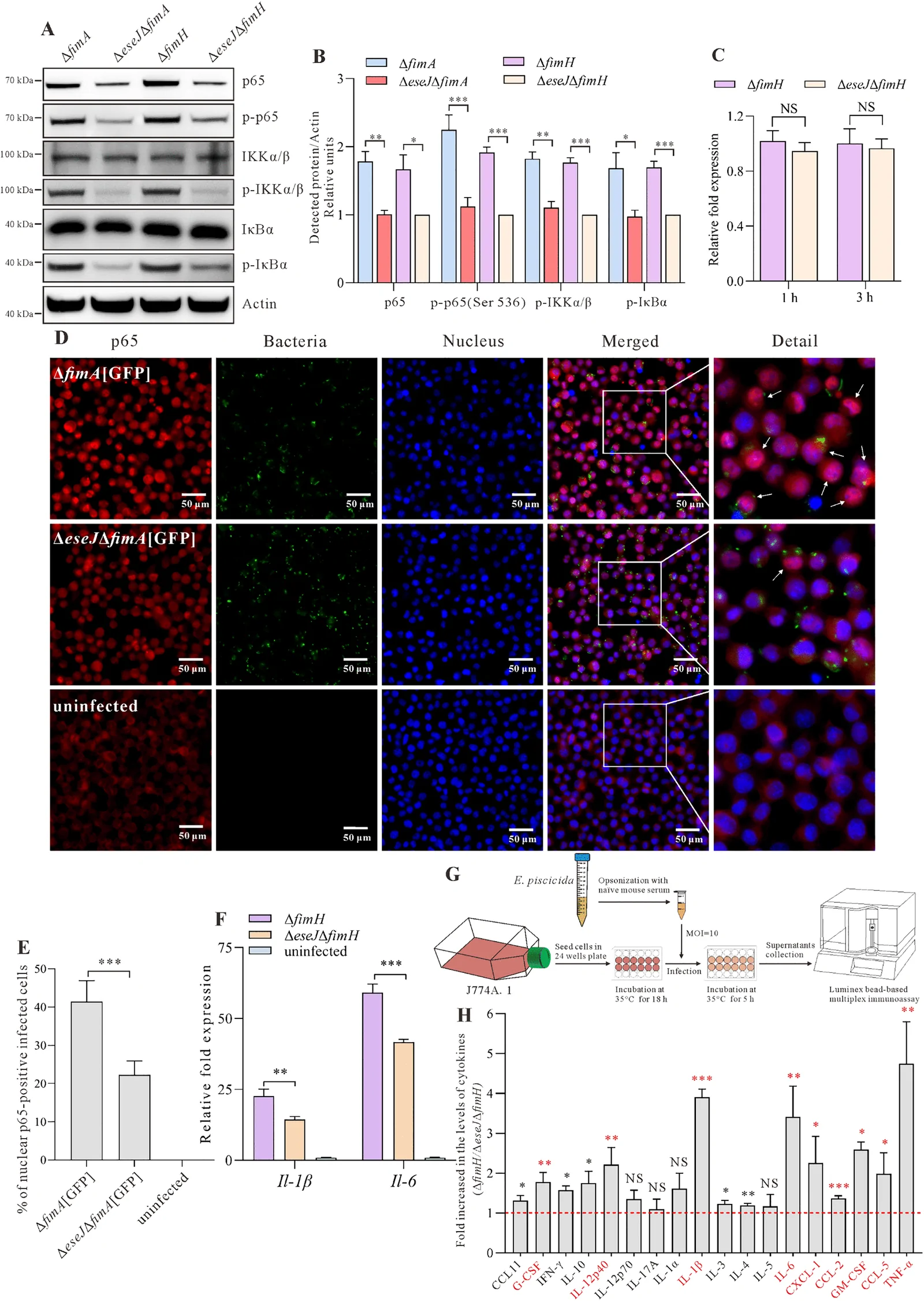
*E. piscicida*-mediated NF-κB activation in J774A.1 macrophages requires EseJ. A. Immunoblot analysis and quantification of total p65, phosphorylated p65 (Ser 536; p-p65), IKKα/β, phosphorylated IKKα/β (p-IKKα/β), IκBα and phosphorylated IκBα (p-IκBα) from J774A.1 macrophages infected with *E. piscicida* Δ*fimA*, Δ*eseJ*Δ*fimA*, Δ*fimH*, and Δ*eseJ*Δ*fimH* strains. Actin was used as the loading control. B. Densitometric quantification of p65, p-p65, p-IKKα/β, and p-IκBα in Fig 1A. Graphs show the relative ratios (Means ± SEM) of the detected proteins from three independent experiments. ***, *p* < 0.001; **, *p* < 0.01; *, *p* < 0.05. C. qRT-PCR analysis of *p65* transcription in J774A.1 macrophages infected with *E. piscicida* Δ*fimH* or Δ*eseJ*Δ*fimH* strain at a MOI of 20 for 1 h and 3 h. The *gapdh* and *β-actin* were used as the reference genes. Means ± SD are shown. NS, not significant. D. Representative confocal images of macrophages infected with GFP-expressing Δ*fimA* and Δ*eseJ*Δ*fimA* strains (green), stained for p65 (red) and nuclei (DAPI, blue). Scale bars: 50 µm. E. Quantification of nuclear p65-positive infected cells (Means ± SD) in Fig 1D. F. qRT-PCR analysis of *Il-1β* and *Il-6* transcription in J774A.1 macrophages infected with Δ*fimH* or Δ*eseJ*Δ*fimH* strain at a MOI of 20 for 3 h. The *gapdh* and *rpl13a* served as the reference genes. Means ± SD are shown. **, *p* < 0.01; *, *p* < 0.05. G. & H. Luminex bead-based multiplex immunoassay of 18 cytokines in supernatants of J774A.1 macrophages infected with Δ*fimH* and Δ*eseJ*Δ*fimH* strains at a MOI of 10 for 5 h (G). Fold changes of cytokine levels in the Δ*fimH*-infected group were normalized to those in the Δ*eseJ*Δ*fimH*-infected group (H). Cytokines regulated by NF-κB are highlighted in red. Means ± SD are shown. ***, *p* < 0.001; **, *p* < 0.01; *, *p* < 0.05; NS, not significant.

To evaluate NF-κB activation, we measured the levels of total and phosphorylated p65, IκBα, and IKKα/β proteins in infected J774A.1 macrophages. Compared with the Δ*fimA* or Δ*fimH* strain, macrophages infected with the Δ*eseJ*Δ*fimA* or Δ*eseJ*Δ*fimH* strain exhibited significantly lower levels of total p65, phosphorylated p65 (p-p65), phosphorylated IKKα/β (p-IKKα/β), and phosphorylated IκBα (p-IκBα) (Fig 1A & 1B). No significant differences were observed in the levels of total IKKα/β and total IκBα/β. Notably, infection with Δ*eseJ*Δ*fimA* or Δ*eseJ*Δ*fimH* strains led to reduced levels of total p65 compared to Δ*fimA* or Δ*fimH* strains (Fig 1A & 1B). However, macrophages infected with these strains showed no significant changes in gene expression of p65 at 1 and 3 hours post-infection (Fig 1C). Nuclear translocation of the p65/p50 dimer is a canonical event in NF-κB activation [20]. To further confirm the role of EseJ in NF-κB activation, J774A.1 macrophages were infected with Δ*fimA*[GFP] and Δ*eseJ*Δ*fimA*[GFP] strains at an MOI of 20 for 3 h, followed by staining for p65 (red), bacteria (green) and DNA (blue) (Fig 1D & 1E). As expected, uninfected J774A.1 macrophages showed almost no nuclear translocation of p65. The percentage of J774A.1 macrophages with nuclear translocation of p65 was significantly higher in cells infected with the Δ*fimA*[GFP] strain compared with cells infected with the the Δ*eseJ*Δ*fimA*[GFP] strain. These data suggest that during *E. piscicida* infection, EseJ promotes p65 stability at the protein level but does not alter its transcription.

The activation of NF-κB signaling leads to increased production of various proinflammatory cytokines [39]. Consistent with this, the transcription levels of *Il-1β* and *Il-6* were significantly lower in Δ*eseJ*Δ*fimH*-infected J774A.1 macrophages compared to Δ*fimH*-infected macrophages (Fig 1F). To further confirm the role of EseJ in promoting the production of proinflammatory cytokines, we employed the Luminex bead-based multiplex immunoassay to quantify the levels of 18 cytokines in the culture supernatant of *E. piscicida*-infected J774A.1 macrophages (Fig 1G). Compared with the Δ*eseJ*Δ*fimH* strain, infection with the Δ*fimH* strain significantly increased the secretion levels of various cytokines, including CCL11, IL-10, IL-1β, IL-6, IL-3, IL-4, IL-12 p40, CCL2, CCL5, TNF-α, G-CSF, GM-CSF, CXCL1, and IFN-γ, most of which are regulated by NF-κB signaling (including IL-1β, IL-6, IL-12 p40, CCL2, CCL5, TNF-α, G-CSF, GM-CSF, CXCL1, and CCL11). Notably, the secretion levels of TNF-α, IL-1β, and IL-6 exhibited the biggest increase in the Δ*fimH* strain (Fig 1H).

Together, these findings demonstrate that *E. piscicida* infection activates the NF-κB signaling in J774A.1 macrophages and promotes the production of pro-inflammatory cytokines, and this is mediated by EseJ.

### EseJ is required for *E. piscicida* to suppress K48-linked ubiquitination and proteasomal degradation of p65 in J774A.1 macrophages

**Fig 2:**
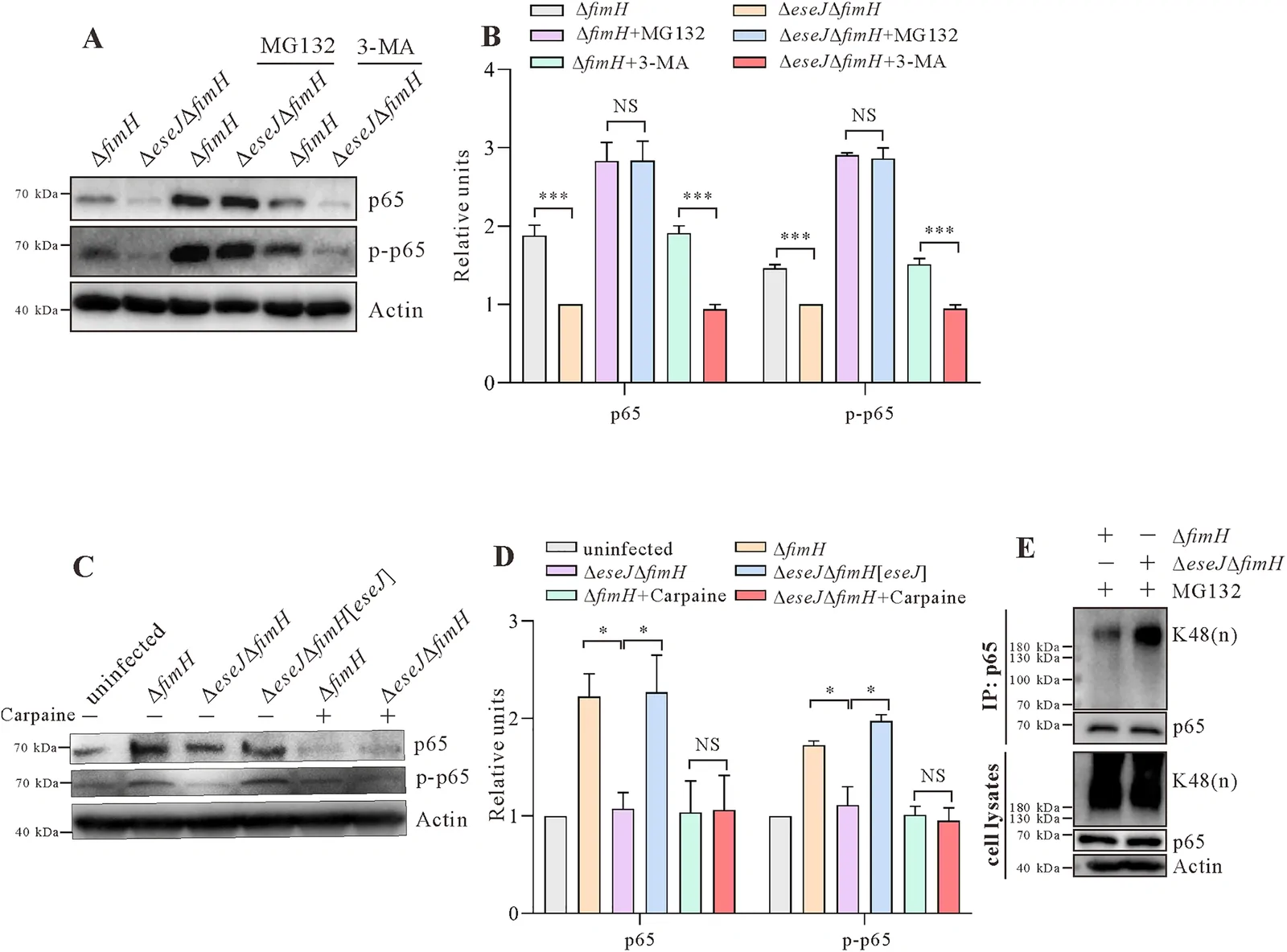
EseJ is required for *E. piscicida* to suppress K48-linked ubiquitination and proteasomal degradation of p65 in J774A.1 macrophages. A. Immunoblot of p65 and p-p65 in J774A.1 macrophages infected with *E. piscicida* Δ*fimH* or Δ*eseJ*Δ*fimH* strain (MOI = 20, for 3 h). J774A.1 macrophages were treated with 20 μM MG132 (proteasome inhibitor) or 5 mM 3-MA (autophagy inhibitor) for 3 h before being infected with the Δ*fimH* or Δ*eseJ*Δ*fimH* strain. Actin was used as the loading control. B. Densitometric quantification of p65 and p-p65 (normalized to actin). Graphs show the relative ratios (Means ± SEM) of the detected proteins from three independent experiments. ***, *p* < 0.001; NS, not significant. C. Immunoblot of p65 and p-p65 in J774A.1 macrophages infected with *E. piscicida* Δ*fimH*, Δ*eseJ*Δ*fimH* or Δ*eseJ*Δ*fimH*[*eseJ*] strain (MOI = 20, for 3 h). The NF-κB inhibitor carpaine (2.0 μM) was added to J774A.1 macrophages 6 h before infection, and remained during infection. Actin was used as the loading control. D. Densitometric quantification of p65 and p-p65 (normalized to actin). Graphs show the relative ratios (Means ± SEM) of the detected proteins from three independent experiments. ***, *p* < 0.001; NS, not significant. E. Immunoprecipitation of endogenous p65, followed by immunoblotting for K48-linked ubiquitin. Cell lysates were collected from J774A.1 macrophages that were infected with *E. piscicida* Δ*fimH* or Δ*eseJ*Δ*fimH* strain (MOI = 20) and treated with 20 μM MG132 for 3 h prior to cell collection. Actin was used as the loading control.

We next investigated how EseJ stabilizes total p65 levels. Two major pathways mediate p65 degradation: the ubiquitin-proteasome system (UPS)-mediated pathway and the autophagy-mediated pathway [40,41]. J774A.1 macrophages were infected with the Δ*fimH* or Δ*eseJ*Δ*fimH* strain, in the absence or the presence of the proteasome inhibitor MG132 or the autophagy inhibitor 3-methyladenine (3-MA) for 3 h. In cells not treated with MG132, Δ*eseJ*Δ*fimH* infection resulted in significantly lower levels of total p65 and phosphorylated p65 compared to Δ*fimH* infection infection (Fig 2A & 2B). Notably, such a difference was abolished in the presence of MG132, but not 3-MA. Infection of J774A.1 macrophages with an *eseJ* complementation strain (Δ*eseJ*Δ*fimH[eseJ]*) restored total and phosphorylated p65 levels, ruling out polar effects from *eseJ* deletion. Finally, treatment with carpaine, an NF-κB inhibitor that promotes p65 degradation, reduced both total and phosphorylated p65 levels and abolished the difference between the two infection conditions (Fig 2C & 2D). These data indicate that EseJ suppresses the proteasomal degradation of p65 rather than its autophagy turnover.

As K48-linked ubiquitination of p65 is responsible for its proteasomal degradation [42], we investigated whether EseJ would interfere with this process. J774A.1 macrophages were infected with either the Δ*fimH* or Δ*eseJ*Δ*fimH* strain, in the presence of MG132 for 3 h. Endogenous p65 was immunoprecipitated, and the K48-ubiquitinated form of p65 was analyzed by immunoblotting. Significantly higher levels of K48-ubiquitinated p65 were detected in J774A.1 macrophages infected with Δ*eseJ*Δ*fimH* strain compared to those infected with Δ*fimH* strain (Fig 2E), suggesting that EseJ inhibits K48-linked ubiquitination and proteasomal degradation of p65.

### EseJ-dependent TAK1 phosphorylation is required for *E. piscicida* to induce NF-κB activation

**Fig 3:**
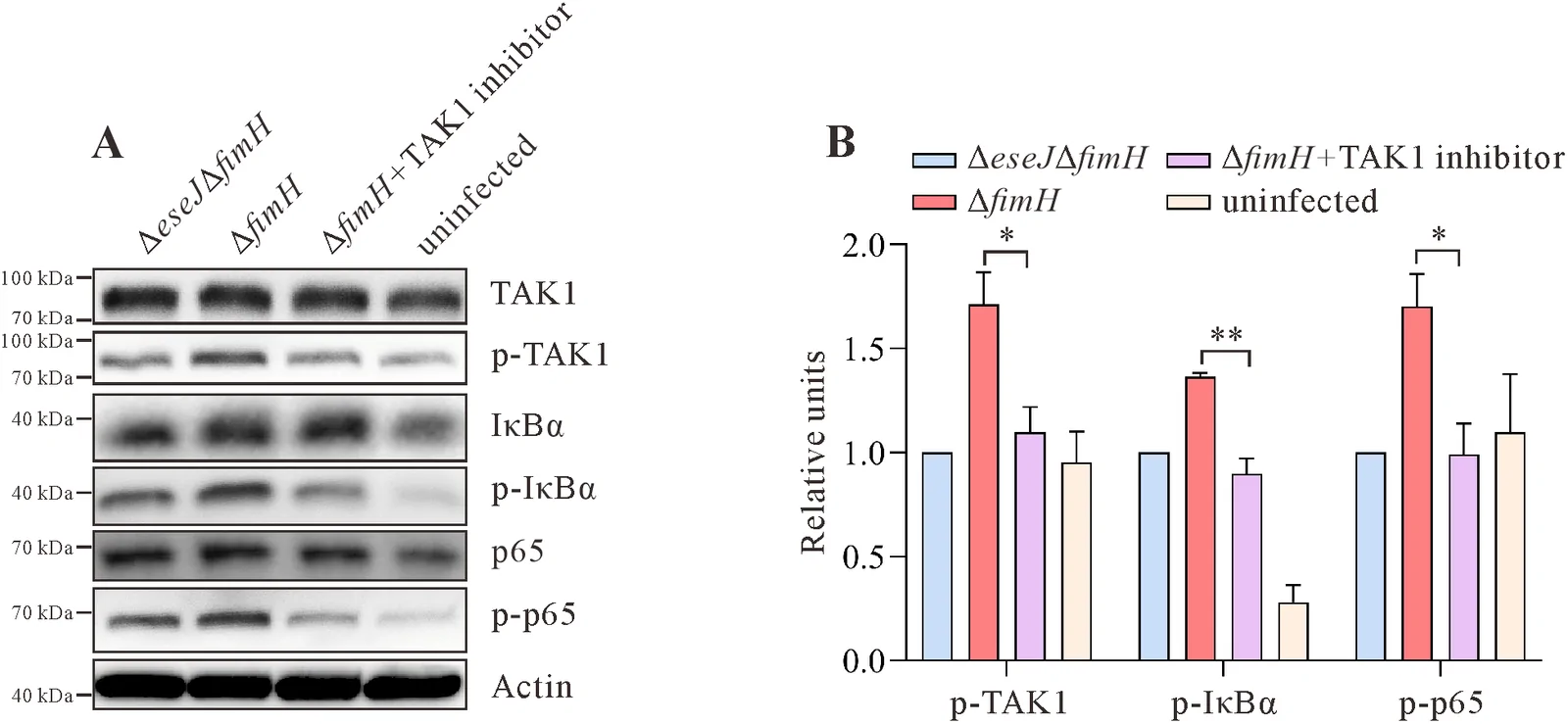
EseJ-dependent TAK1 phosphorylation is required for *E. piscicida* to induce NF-κB activation. A. Immunoblot of TAK1, phosphorylated TAK1 (p-TAK1), IκBα, p-IκBα, p65, and p-p65 in J774A.1 macrophages infected with *E. piscicida* Δ*fimH* or Δ*eseJ*Δ*fimH* strain (MOI = 20, for 3 h). J774A.1 macrophages were pretreated with the TAK1 inhibitor TAKinib for 1 h prior to infection with the Δ*fimH* strain. Actin was used as the loading control. B. Densitometric quantification of p-TAK1, p-IκBα and p-p65 (normalized to actin). Graphs show the relative ratios (Means ± SEM) of the detected proteins from three independent experiments. **, *p* < 0.01; *, *p* < 0.05.

TAK1 is a key mediator of the NF-κB signaling [43]. Upon activation, TAK1 phosphorylates IκB kinase (IKK), which then induces the phosphorylation of IκB, thereby activating NF-κB [27]. We asked whether EseJ would activate the IKK-IκB-NFκB signaling pathway via TAK1. J774A.1 macrophages were pre-treated with or without the 50 μM TAKinib (TAK1 inhibitor) for 1 h, followed by infection with the Δ*fimH* strain at an MOI of 20 for 3 h. The Δ*eseJ*Δ*fimH* strain was also used for infection. As shown in Fig 3A & 3B, cells infected with Δ*eseJ*Δ*fimH*, and cells infected with Δ*fimH* and pretreated with TAK inhibitor, showed significantly lower levels of phosphorylated TAK1 (p-TAK1), phosphorylated IκBα (p-IκBα) and phosphorylated p65 (p-p65) compared to cells infected with Δ*fimH*. These results suggest that EseJ mediates *E. piscicida*-induced TAK1-IKK-IκB-NF-κB signaling pathway.

### The N-terminal region of EseJ binds to p65 and prevents K48-linked p65 ubiquitination and proteasomal degradation

**Fig 4:**
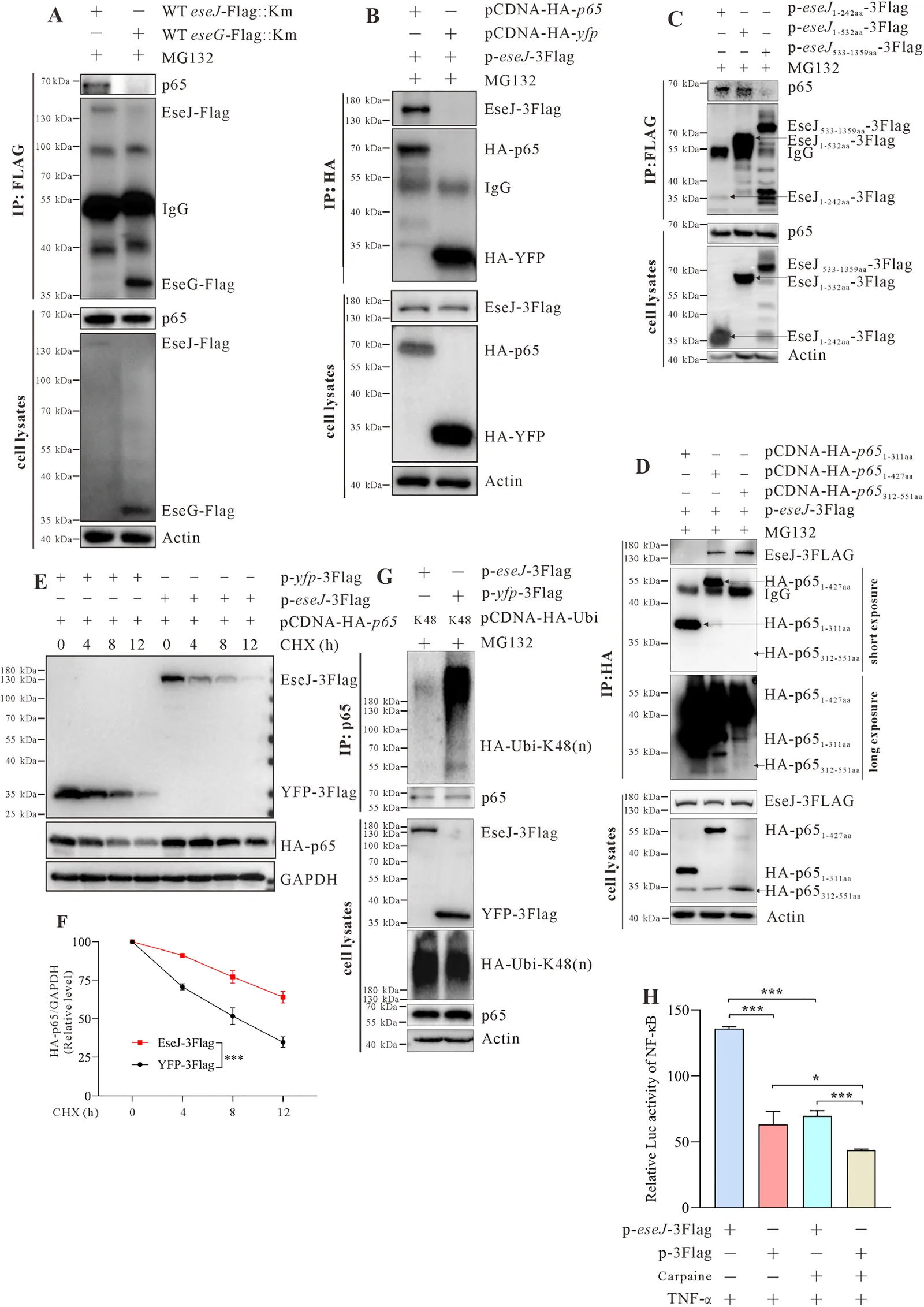
The N-terminus of EseJ binds to p65 and prevents K48-linked p65 ubiquitination and proteasomal degradation. A. Immunoprecipitation of endogenous p65 from cell lysates of J774A.1 macrophages that were infected with *E. piscicida* WT *eseJ*-Flag::Km or WT *eseG*-Flag::Km strain (MOI = 10) and treated with 20 μM MG132 for 3 h. Immunoblot was performed to detect the interaction between endogenous p65 and translocated EseJ by WT strain in J774A.1 cell lysates. Actin was used as the loading control. B. Immunoprecipitation of HA-tagged proteins from cell lysates of HEK293T cells that were co-transfected with pCDNA-HA*-p65*/pCDNA-HA-*yfp* and p-*eseJ*-3Flag, and treated with 20 μM MG132 for 8 h. Immunoblot was performed to detect the interaction between HA-p65 and EseJ-3Flag. Actin was used as the loading control. C. Immunoprecipitation of Flag-tagged proteins from cell lysates of HEK293T cells that were transfected with p-*eseJ*_1-242 aa_-3Flag, p-*eseJ*_1-532 aa_-3Flag, and p-*eseJ*_533-1359 aa_-3Flag, followed by treatment with 20 μM MG132 for 8 h. Immunoblot was conducted to detect the interaction between endogenous p65 and exogenous regions of EseJ. Actin was used as the loading controls. D. Co-immunoprecipitation of p65 domains with EseJ. Lysates from HEK293T cells expressing pCDNA-HA-*p65*_1–311 aa_, pCDNA-HA-*p65*_1–427 aa_ or pCDNA-HA-*p65*_312–551 aa_ plus p-*eseJ*-3Flag for 16 h. The cells were treated with 20 μM MG132 for 8 h before being immunoprecipitated with anti-HA and immunoblotted for EseJ-Flag and actin (the loading control).E. Immunoblot of HA-p65 using cell lysates from HEK293T cells co-transfected with pCDNA-HA-*p65* plus p-*eseJ*-3Flag/p-*yfp*-3Flag plasmids. After 18 h of culture, transfected cells were treated with 50 μM CHX for 0 h, 4 h, 8 h, and 12 h before collection. GAPDH was used as the loading control. F. Densitometric quantification of HA-p65 normalized to GAPDH. Graphs show the relative ratios (Means ± SEM) of the detected proteins from three independent experiments. ***, *p* < 0.001. G. Immunoprecipitation of endogenous p65 from cell lysates of HEK293T cells that were co-transfected with p-*yfp*-3Flag/p-*eseJ*-3Flag plus pCDNA-HA-Ubi-K48, and treated with 20 μM MG132 for 8 h prior to cell collection. Immunoblot was conducted to detect the level of K48-ubiquitinated p65. Actin was used as the loading control. H. Dual-luciferase reporter assays of NF-κB activation. HEK293T cells were co-transfected with NF-κB reporter (p-NF-κB-Luc and pRL-TK) and p-*eseJ*-3Flag or empty p-3Flag vector plasmids for 16 h, and treated with 15.0 ng/ml TNF-α and 2.0 μM carpaine (or not) for 8 h before sampling for luciferase assays.

The observation that EseJ suppresses p65 proteasomal degradation raises the possibility that EseJ may bind to p65. To test this, J774A.1 macrophages were infected with wild-type *E. piscicida* expressing EseJ-Flag

(WT *eseJ-*Flag::Km) or EseG-Flag (WT *eseG-*Flag::Km) on its chromosome. EseG is another T3SS effector [10]. At 3 h post infection, we immunoprecipitated EseJ-Flag and EseG-Flag with an anti-Flag antibody, and probed for p65. As shown in Fig 4A, p65 was specifically pulled down by EseJ-Flag, but not EseG-Flag.

EseJ functions as both a T3SS effector and a T3SS regulator [16,38]. It is possible that during *E. piscicida* infection, other T3SS effectors or EseJ-regulated bacterial proteins mediate the interaction of EseJ with p65. To rule out this possibility, EseJ-3Flag was co-expressed with either HA-p65 or HA-YFP (negative control) in HEK293T cells. Following co-immunoprecipitation (Co-IP) with anti-Flag antibodies, we observed that EseJ-3Flag selectively pulled down HA-p65, whereas no interaction was detected with HA-YFP (Fig 4B). These results indicate that EseJ specifically interacts with p65. To map the p65-binding domain of EseJ, HEK293T cells were transfected with C-terminally 3 × Flag-tagged truncation fragments. These constructs contained the N-terminal regions (aa 1–242 and 1–532) and the C-terminal region (aa 533–1359) of EseJ, respectively. As shown in Fig 4C, EseJ_1-242 aa_-3Flag and EseJ_1-532 aa_-3Flag, but not EseJ_533-1359 aa_-3Flag, pulled down endogenous p65, suggesting that the p65-interacting domain of EseJ is located within the first 242 amino acids of its N-terminal region. To map the EseJ-binding domain of p65, HEK293T cells were transfected with N-terminally HA-tagged truncation constructs containing amino acids 1–311, 1–427, or 312–551 of p65. Co-IP showed that HA-p65_1–427 aa_ and HA-p65_312–551 aa_, but not HA-p65_1–311 aa_, pulled down EseJ-3Flag (Fig 4D), suggesting that the EseJ-interacting domain of p65 is located within the C-terminal region (aa 312–551) of p65.

We next investigated whether ectopic expression of EseJ stabilizes p65 by inhibiting its degradation. HEK293T cells were co-transfected with HA-p65 and either EseJ-3Flag or YFP-3Flag (negative control). To monitor protein turnover, cells were treated with cycloheximide (CHX) to arrest *de novo* protein synthesis [44]. We observed that EseJ-3Flag significantly inhibited the degradation of HA-p65 in a time-dependent manner compared to the YFP control (Fig 4E & 4F). Densitometric analysis revealed that the p65 half-life was 8.10 h in cells expressing YFP compared to 20.43 h in those expressing EseJ, and the stability of the HA-p65 between these two groups is significantly different (Fig 4F). To determine if this stabilization resulted from impaired proteasomal targeting, we co-transfected cells with HA-Ubi-K48 and EseJ-3Flag or YFP-3Flag in the presence of the proteasome inhibitor MG132. Co-immunoprecipitation analysis revealed that EseJ-3Flag expression led to a marked reduction in K48-linked ubiquitination of p65 (Fig 4G). Collectively, these data support the model that EseJ suppresses p65 degradation by interfering with its K48-linked ubiquitination.

To further confirm that EseJ activates NF-κB signaling through modulating p65 protein levels, we performed a dual-luciferase reporter assay in HEK293T cells in the absence or presence of the NF-κB inhibitor carpaine. Cells were co-transfected with p-NF-κB-Luc (expressing firefly luciferase under control of NF-κB promoter), pRL-TK (expressing Renilla luciferase constitutively), and either p-*eseJ*-3Flag or empty p-3Flag vector, with or without carpaine treatment. TNF-α (15 ng/ml) was also added to stimulate NF-κB activation [29], which was expressed as the ratio of NF-κB-driven luciferase activity to Renilla luciferase activity. As shown in Fig 4H, *p*-*eseJ*-3Flag transfection nearly tripled relative NF-κB activity compared to the empty p-3Flag vector control. Carpaine treatment attenuated NF-κB activation across both groups, although cells expressing p-*eseJ*-3Flag maintained marginally higher activation levels than those transfected with the empty vector. These results suggest that EseJ induces NF-κB activation by suppressing p65 degradation.

Taken together, EseJ binds to p65 through its N-terminal 1–242aa, and prevents K48-linked p65 ubiquitination and proteasomal degradation. This ultimately induces NF-κB activation.

### EseJ also binds to TAK1 and TRAF6 and enhances TRAF6-mediated K27-linked ubiquitination of TAK1

**Fig 5:**
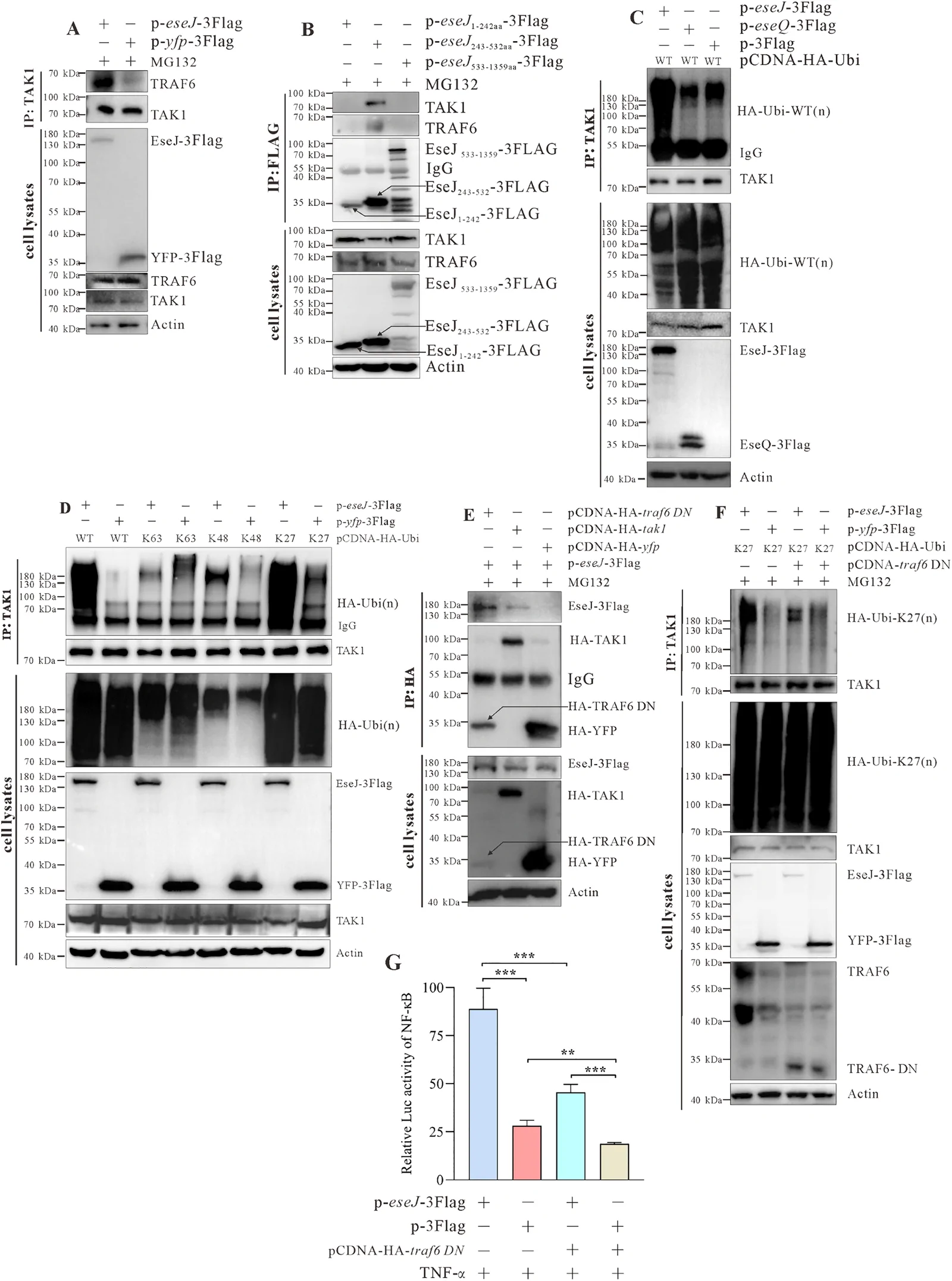
EseJ also binds to TAK1 and TRAF6 and enhances TRAF6-mediated K27-linked ubiquitination of TAK1. A. Immunoprecipitation of endogenous TAK1 from cell lysates of HEK293T cells that were transfected with p-*yfp*-3Flag or p-*eseJ*-3Flag for 16 h, and treated with 20 μM MG132 for another 8 h prior to cell collection. Immunoblot was conducted to detect the interaction level between endogenous TAK1 and endogenous TRAF6 in different cell lysates. Actin was used as the loading control. B. Co-immunoprecipitation of EseJ domains with TAK1 and TRAF6. Lysates from HEK293T cells expressing p-*eseJ*_1-242 aa_-3Flag, p-*eseJ*_243-532 aa_-3Flag, or p-*eseJ*_533-1359 aa_-3Flag treated with 20 μM MG132 (8 h) were immunoprecipitated with anti-Flag and immunoblotted for endogenous TAK1, TRAF6, and actin (the loading control). C. Immunoprecipitation of endogenous TAK1 from cell lysates of HEK293T cells that were co-transfected with p-3Flag/p-*eseQ*-3Flag/p-*eseJ*-3Flag, and HA-Ubi-WT plasmids for 16 h, and treated with 20 μM MG132 for another 8 h prior to cell collection. Immunoblotting was conducted to detect the complete ubiquitination of endogenous TAK1. Actin was used as the loading control. D. Immunoprecipitation of endogenous TAK1 from cell lysates of HEK293T cells that were co-transfected with p-*yfp*-3Flag/p-*eseJ*-3Flag plus ubiquitin plasmids HA-Ubi-WT, HA-Ubi-K63, HA-Ubi-K48 or HA-Ubi-K27 for 16 h, and treated with 20 μM MG132 for another 8 h before cell collection. Immunoblotting was conducted to detect ubiquitination of endogenous TAK1 in cell lysates. Actin was used as the loading controls. E. Immunoprecipitation of HA-tagged proteins from cell lysates of HEK293T cells that were co-transfected with pCDNA-HA-*traf6* DN/ pCDNA-HA-*tak1*/pCDNA-HA-*yfp* plus p-*eseJ*-3Flag plasmids for 16 h, and treated with 20 μM MG132 for another 8 h before cell collection. Immunoblotting was conducted to detect the interaction between HA-TRAF6 DN/HA-TAK1 and EseJ-3Flag were detected from cell lysates. Actin was used as the loading controls. F. Immunoprecipitation of endogenous TAK1 from cell lysates of HEK293T cells that were co-transfected with p-*yfp*-3Flag/p-*eseJ*-3Flag plus ubiquitin plasmids HA-Ubi-K27 and pCDNA-*traf6* DN (or not) for 16 h, and treated with 20 μM MG132 for another 8 h before cell collection. Immunoblotting was conducted to detect the K27-linked ubiquitination of endogenous TAK1 in cell lysates. Actin was used as the loading controls.G. Dual-luciferase reporter assays of NF-κB activation. HEK293T cells were co-transfected with NF-κB reporter (p-NF-κB-Luc and pRL-TK) and p-*eseJ*-3Flag or empty p-3Flag vector plasmids with or without pCDNA-*traf6 DN* plasmid for 16 h, and treated with 15.0 ng/ml TNF-α for another 8 h before luciferase assays.

We next investigated whether EseJ associates with the TAK1-TRAF6 signaling complex. HEK293T cells were transfected with EseJ-3Flag or YFP-3Flag (negative control), and endogenous TAK1 was analyzed by immunoprecipitation. We observed that the presence of EseJ-3Flag significantly enhanced the recruitment of endogenous TRAF6 to TAK1 compared to the YFP control (Fig 5A). To locate the specific binding domains, we used a series of EseJ truncation fragments (aa 1–242, 243–532, and 533–1359). While the aa 1–242 and 533–1359 fragments failed to associate with the complex, the EseJ_243-532 aa_ construct retained the ability to bind both endogenous TAK1 and TRAF6 (Fig 5B). These data demonstrate that the region containing aa 243–532 is required for the interaction of EseJ with the TAK1-TRAF6 complex.

TAK1 is modified with non-degradative K63-linked and K27-linked polyubiquitin chains, which serve as essential molecular scaffolds to recruit and activate NF-κB signaling [29,45]. We hypothesized that the physical interaction between EseJ and TAK1 might promote TAK1 ubiquitination. To test this, HEK293T cells were transfected with HA-Ubi (WT) plus EseJ-3Flag, EseQ-3Flag, or p-3Flag (empty vector). EseQ is another T3SS effector in *E. piscicida* [14]. We immunoprecipitated endogenous TAK1 and examined TAK1 ubiquitination by immunoblotting. The levels of ubiquitinated TAK1 were significantly higher in cells transfected with EseJ-3Flag compared to those transfected with EseQ-3Flag or p-3Flag (Fig 5C). To further delineate the specific ubiquitin linkages promoted by EseJ, we co-transfected HEK293T cells with EseJ-3Flag and various HA-tagged ubiquitin mutants (WT, K63-only, K48-only, and K27-only). Immunoprecipitation of endogenous TAK1 revealed a marked increase in K27-linked polyubiquitination of TAK1 in EseJ-transfected cells (Fig 5D). In contrast, the levels of K63- and K48-linked ubiquitination remained comparable between EseJ-3Flag and YFP-3Flag transfected cells. Collectively, these results demonstrate that EseJ specifically enhances the K27-linked polyubiquitination of TAK1.

TRAF6 is a known E3 ligase that catalyzes the formation of K27-linked polyubiquitin chains on TAK1 [29]. To determine whether TRAF6 is required for EseJ-mediated TAK1 ubiquitination, we used a dominant-negative TRAF6 construct (TRAF6-DN). The TRAF6-DN construct encodes aa 289–522, encompassing the conserved coiled-coil and TRAF-C domains. It lacks E3 ligase activity and acts as a potent inhibitor of TRAF6 signaling by blocking TRAF6 oligomerization and the interaction of TRAF6 with upstream signaling adaptors [46]. We first confirmed if EseJ would interact with TRAF6-DN by co-expressing EseJ-3Flag with HA-tagged TAK1 or HA-tagged TRAF6-DN. Co-IP revealed that EseJ-3Flag interacted with both HA-TAK1 and HA-TRAF6-DN, but not the HA-YFP control (Fig 5E). We then determined if TRAF6-DN would prevent EseJ-induced K27-linked ubiquitination of TAK1. HEK293T cells were co-transfected with HA-Ubi-K27 and either EseJ-3Flag or YFP-3Flag in the presence or absence of TRAF6-DN. As shown in Fig 5F, Immunoprecipitation of endogenous TAK1 revealed that ectopic expression of TRAF6-DN reduced the levels of K27-linked ubiquitination of TAK1 in cells transfected with EseJ-3Flag (Fig 5F). These results suggest that EseJ promotes the K27-linked polyubiquitination of TAK1 in a TRAF6-dependent manner.

To validate that TRAF6 is required for EseJ-induced NF-κB activation, we also performed the dual-luciferase reporter assay in HEK293T cells. Cells were co-transfected with p-NF-κB-Luc, pRL-TK, p-*eseJ*-3Flag or empty p-3Flag vector, with or without pCDNA-HA-TRAF6-DN, and treated with TNF-α. Expression of HA-TRAF6 DN significantly reduced NF-κB activation in both p-*eseJ*-3Flag and empty vector groups (Fig 5G). Notably, the fold-increase in NF-κB activity between p-*eseJ*-3Flag and empty vector groups was markedly reduced upon co-expression of HA-TRAF6 DN, suggesting that EseJ activates NF-κB pathway in a TRAF6 dependent manner.

Collectively, these data demonstrate that EseJ associates with both TAK1 and TRAF6 through its central domain (aa 243–532). By acting as a molecular scaffold, EseJ facilitates TRAF6-TAK1 interaction, thereby enhancing K27-linked polyubiquitination of TAK1 and activating the NF-κB signaling pathway.

### EseJ is required for *E. piscicida* to induce NF-κB activation and the production of pro-inflammatory cytokines *ex vivo* and *in vivo*

**Fig 6:**
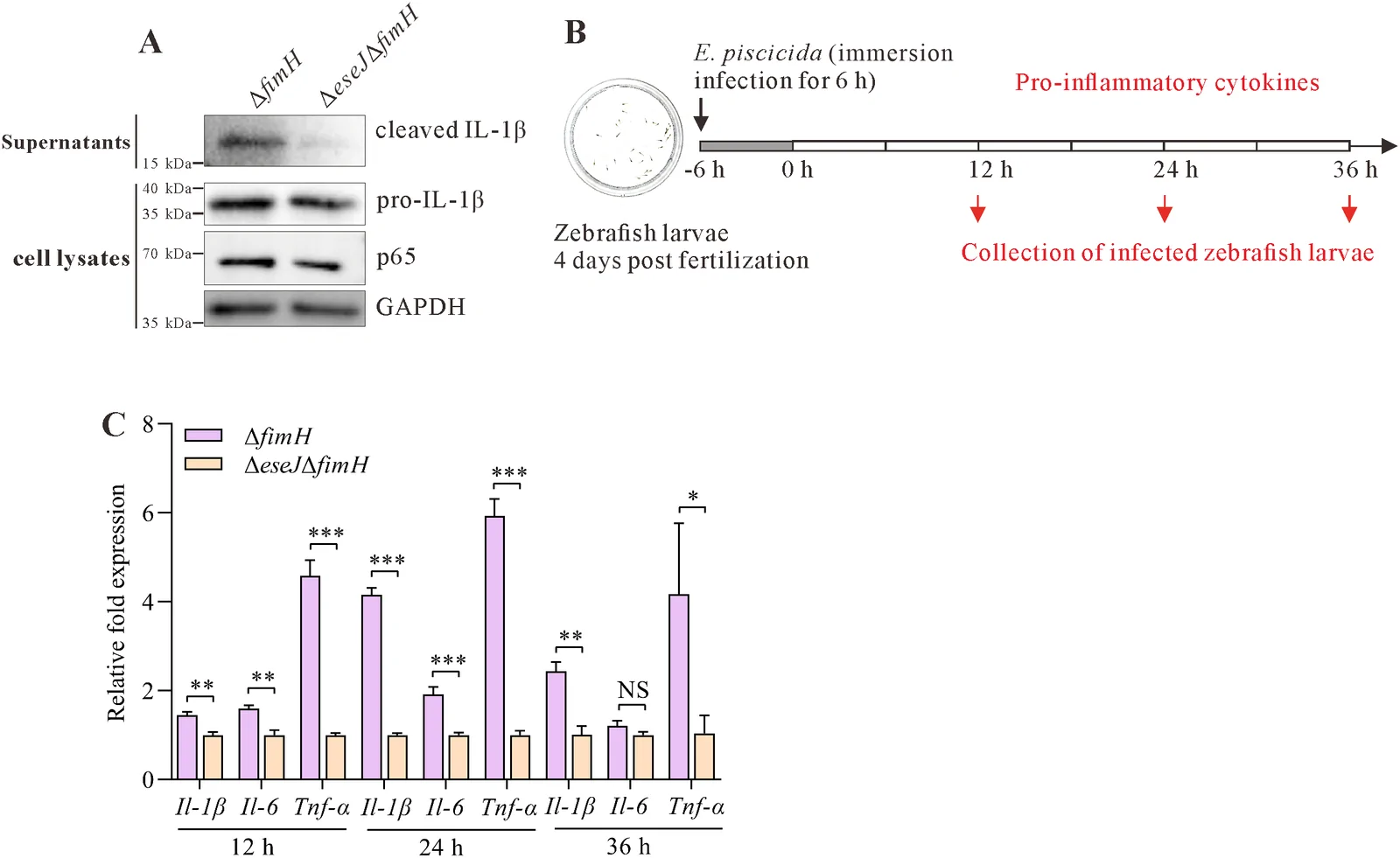
EseJ is required for *E. piscicida* to induce NF-κB activation and the production of pro-inflammatory cytokines *ex vivo* and *in vivo.* A. Immunoblots of p65, pro-IL-1β, and cleaved IL-1β in leukocytes infected with *E. piscicida* Δ*fimH* or Δ*eseJ*Δ*fimH* (MOI = 20, for 3 h). GAPDH was used as the loading control. B. Schematic illustration of immersion challenge with *E. piscicida* Δ*fimH* or Δ*eseJ*Δ*fimH* (5 × 10⁸ CFU/mL) and sampling time-points (12/24/36 hpi). C. qRT-PCR analysis of *Il-1β*, *Il-6*, and *Tnf-α* transcription from infected zebrafish larvae. The *gapdh* and *actin* genes were used as the reference genes. Red font indicates the time points at which infected zebrafish were collected to detect pro-inflammatory cytokines. Means ± SD are shown. ***, *p* < 0.001; **, *p* < 0.01; *, *p* < 0.05; NS, not significant.

Finally, we sought to determine whether *E. piscicida* uses EseJ to induce NF-κB activation and pro-inflammatory cytokine production in primary leukocytes and *in vivo*. Primary leukocytes isolated from the head kidneys of healthy yellow catfish (*Pelteobagrus fulvidraco*) were infected with Δ*fimH* or Δ*eseJ*Δ*fimH* (MOI = 20). At 5 h post-infection, Δ*eseJ*Δ*fimH* induced much lower levels of total p65, pro-IL-1β, and cleaved IL-1β compared to Δ*fimH* (Fig 6A). To evaluate the role of EseJ *in vivo*, we utilized a zebrafish larval infection model. Four-day-old larvae were challenged by immersion with Δ*fimH* or Δ*eseJ*Δ*fimH* strain (5 × 10^8^ CFU/mL), and the expression of *Il-1β*, *Il-6*, and *Tnf-α* was examined at 12, 24, and 36 hours post-infection (Fig 6B). Consistent with our *in vitro* findings (Fig 1F), the expression levels of *Il-1β*, *Il-6*, and *Tnf-α* were significantly lower in larvae infected with Δ*eseJ*Δ*fimH* at each time point examined except for *il*-6 at 36 hpi (Fig 6C). Collectively, these data demonstrate that EseJ is required for *E. piscicida*-induced NF-κB activation and the subsequent pro-inflammatory responses.

## Discussion

While an early report established that *E. piscicida* induces NF-κB-dependent gene expression through a T3SS-dependent mechanism [37], the underlying biochemical basis has not been identified. Our data bridge this gap by identifying EseJ as the primary effector driving this response. Unlike many T3SS effectors that act as inhibitors, our results demonstrate that EseJ functions as a potent activator, providing a mechanistic explanation for the sustained inflammatory signaling observed during *E. piscicida* infection.

The NF-κB signaling pathway serves as a central hub for the coordination of both innate and adaptive immune responses. Consequently, Gram-negative pathogens have evolved diverse strategies to either suppress or promote NF-κB activation to facilitate their survival [32]. For instance, enteropathogenic *Escherichia coli* (EPEC) utilizes a suite of T3SS effectors to bi-directionally modulate this pathway: while NleC and NleE function as potent inhibitors [47–49], EspT and NleF actively drive NF-κB stimulation [50,51]. A similar paradigm is observed in *Shigella flexneri*, which employs OspI to dampen inflammatory signaling [52] while leveraging IpgB2 and OspB to promote inflammation [53]. Analogous to these pathogens, our study identifies EseJ as a critical activator of NF-κB and its downstream IL-1β production in *E. piscicida*. This finding reconciles the contradictive observations in the Δ*eseJ* strain in our previous study [38], where Δ*eseJ* significantly activates caspase-1/8 and GSDMD to drive PANoptosis, but leads to reduced IL-1β secretion. However, whether EseJ-driven inflammation ultimately promotes bacterial clearance or causes tissue damage *in vivo* warrants further study. Interestingly, the pathogenesis of *E. piscicida* is further complicated by the presence of effectors like Trx2, which has been shown to block NF-κB signaling [14]. This suggests that *E. piscicida* may maintain a temporal and spatial control over host inflammation. Our future work will focus on defining how the interplay between EseJ and Trx2 dictates the signaling outcomes in different cell types, such as intestinal epithelial cells versus myeloid cells.

**Fig 7:**
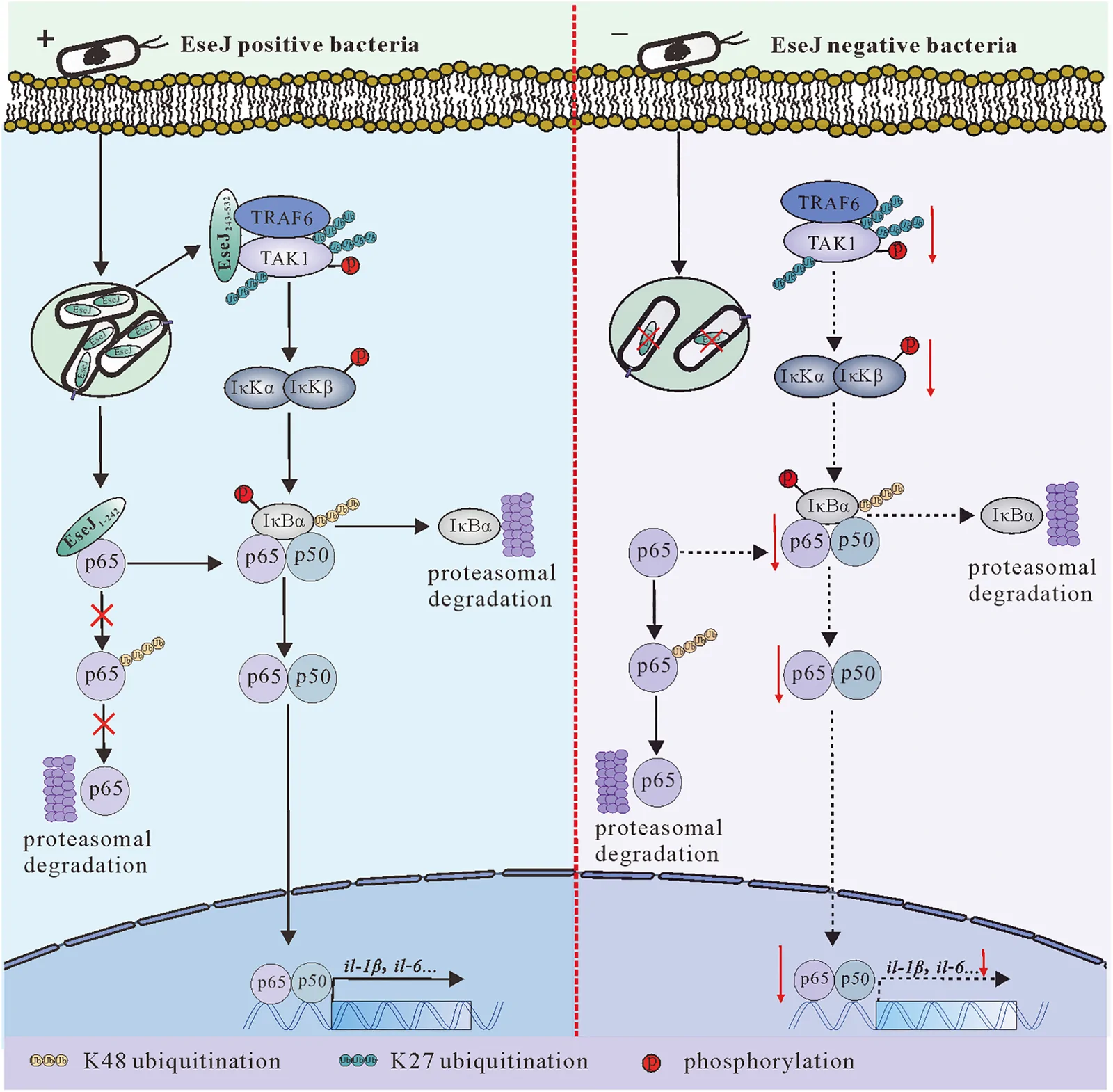
A proposed model illustrating how *E. piscicida* EseJ activates NF-κB. The schematic model illustrates the dual mechanism by which the type III secretion system (T3SS) effector EseJ stimulates NF-κB activation following *Edwardsiella piscicida* infection. First, EseJ binds to p65, preventing K48-linked ubiquitination and proteasomal degradation of p65. Second, EseJ functions as a scaffold to bridge TRAF6 and TAK1, promoting the assembly of TRAF6-TAK1 complex and TRAF6-mediated K27-linked ubiquitination of TAK1, which triggers TAK1 phosphorylation. Phosphorylated TAK1 subsequently activates the IKKα/β complex, leading to IκBα degradation. This dual mechanism promotes p65 nuclear translocation and the transcription of pro-inflammatory cytokine genes, including *Il-1β*, and *Il-6*, *etc*.

Our previous study revealed EseJ as a bacterial transcriptional regulator involved in suppressing the type 1 fimbrial operon [16]. However, the present study expands the functional repertoire of EseJ, identifying it as a potent T3SS effector that directly manipulates host innate immune signaling. By utilizing ectopic expression in HEK293T cells to decouple its bacterial regulatory roles from its effector functions, we demonstrated that EseJ serves as a key molecular scaffold that targets multiple components of the NF-κB signaling cascade (Fig 7). A striking feature of EseJ is its domain-specific function, which allows it to simultaneously enhance the stability of the NF-κB machinery and accelerate its activation. The N-terminal region (aa 1–242) appears to shield the p65 subunit, preventing its K48-linked polyubiquitination and subsequent proteasomal turnover. Concurrently, the central domain (aa 243–532) facilitates the assembly of a stable TRAF6-TAK1 signaling complex, promoting TRAF6-dependent K27-linked polyubiquitination and the subsequent phosphorylation of TAK1. The scaffolding strategy used by *E. piscicida* EseJ mirrors that of other highly adapted pathogens. For example, the *Helicobacter pylori* CagA (T4SS effector) directly associates with TAK1, enhancing its ability to activate IKK and downstream pro-inflammatory signaling [54]. It remains to be determined whether EseJ forms distinct complexes with p65 and TAK1/TRAF6 or assembles into a larger complex.

EseJ is among the few T3SS effectors characterized to date that interact with the core machinery of the NF-κB signaling. This distinguishes EseJ from well-studied inhibitors like *Yersinia* YopJ, which binds and acetylates the activation loop of IKKβ to block signaling [55], or EPEC NleC, which degrades the host co-activator p300 to prevent the essential acetylation of the p65 subunit [49]. In contrast to the inhibitory role of these effectors, EseJ exhibits a unique scaffolding-like function; its interaction with p65, TAK1, and TRAF6 specifically drives the activation of NF-κB signaling. While other effectors, such as EPEC EspT and NleF, *S. flexneri* IpgB2 and OspB, and *S. Typhimurium* SipA [56], are known to stimulate NF-κB, it remains largely unclear whether they achieve this through interaction with NF-κB signaling molecules.

*E. piscicida* EseJ share high homologies with proteins in diverse Gram-negative pathogens (S1 Table). The most striking homology is observed in EseJ of *Edwardsiella ictaluri* (85.4% similarity), a closely related Gram-negative pathogen that also employs T3SS and T6SS to subvert host immunity [57]. The high similarity of these proteins suggests that the manipulation of the NF-κB axis may be a conserved virulence strategy across the *Edwardsiella* genus, although this awaits further investigations. Beyond *Edwardsiella* species, we identified similarities between EseJ and several characterized or putative T3SS effectors in other bacteria. These include the T3SS effector of *Chromobacterium amazonense* (57.6% similarity) and the BopA or BopA-like proteins found in *Shewanella baltica* (39.6% similarity), *Vibrio ostreicida* (35.7% similarity), and *Vibrio hepatarius* (33.2% identity). Whether these EseJ-like proteins have a similar effect on NF-κB signaling remains to be further studied.

In conclusion, we demonstrate that EseJ is the first T3SS effector characterized to date that targets both TAK1 and p65 to promote NF-κB-dependent inflammatory responses. Whether EseJ-driven inflammation promotes bacterial clearance or tissue damage *in vivo* requires further investigation. Given the high homology of EseJ between *E. piscicida* and *E. ictaluri*, our findings suggest a conserved role for EseJ in inducing NF-κB-dependent inflammatory responses across these species. This finding redefines our understanding of *E. piscicida* pathogenesis.

## Materials and methods

### Ethics statement

Fish experiments in this study were conducted at the Institute of Hydrobiology, Chinese Academy of Sciences, in accordance with European Union guidelines for handling of laboratory animals (2010/63/EU) and the protocol employed was approved by the Ethics Committee for Animal Experiments of the Institute of Hydrobiology, Chinese Academy of Sciences (No. IHB/LL/2022030).

### Bacterial strains, cells and cultivation

*E. piscicida* strain PPD130/91, previously designated as *E. tarda* PPD130/91 [2], was used in this study. All bacterial strains and plasmids employed in this study are detailed in Table 1. *E. piscicida* strains were cultured in tryptic soy broth (TSB) at 28°C. Antibiotics were added to the culture medium at the following concentrations when necessary: 12.5 μg/mL colistin (Col; Sigma), 50 μg/mL gentamicin (Gem; Sigma), 100 μg/mL ampicillin (Amp; Sigma).

**Table 1:** Strains and plasmids used in this study.

Strain or plasmid	Description and/or genotype	Reference or source
Bacteria		
*E. piscicida*		
Δ*fimA*	in-frame deletion 1–178 aa of FimA, *E. piscicida* PPD130/91	[16]
Δ*fimH*	in-frame deletion 1–357 aa of FimH, *E. piscicida* PPD130/91	[16]
Δ*eseJ*Δ*fimA*	in-frame deletion of EseJ and FimA, *E. piscicida* PPD130/91	[16]
Δ*eseJ*Δ*fimH*	in-frame deletion of EseJ and FimH, *E. piscicida* PPD130/91	[16]
Δ*eseJ*Δ*fimH[eseJ]*	Δ*eseJ*Δ*fimH* with pACYC-*eseJ*-HA, Tet^r^	This study
Δ*fimA*[GFP]	Δ*fimA* with pFPV25.1	[38]
Δ*eseJ*Δ*fimA*[GFP]	Δ*eseJ*Δ*fimA* with pFPV25.1	[38]
^WT^ *eseJ*^-Flag::Km^	*E. piscicida* PPD130/91 with chromosomal *eseJ* tagged with Flag at its C-terminus, Col^r^, Km^r^	This study
^WT^ *eseG*-Flag::Km	PPD130/91 with chromosomal *eseG* tagged with Flag at its C-terminus, Col^r^, Km^r^	This study
*E. coli*		
DH5α	α complementation	Stratagene
Plasmids		
pFPV25.1	Derivative of pBR322, *gfpmut3A* under constitutive promoter	[58]
p-3Flag	CMV promoter, Amp^r^ Neo^r^	Invitrogen
p-*yfp*-3Flag	p-3 × Flag with *yfp*, Amp^r^	[59]
p-*eseJ*-3Flag	p-3 × Flag with *eseJ*, Amp^r^	This study
p-*eseQ*-3Flag	p-3 × Flag with *eseQ*, Amp^r^	[60]
pCDNA-HA-Ubi-WT	pCDNA3.1 with HA-Wild Type ubiquitin, Amp^r^	[59]
pCDNA-HA-Ubi-K63	pCDNA3.1 with HA-K63 only ubiquitin, Amp^r^	[59]
pCDNA-HA-Ubi-K48	pCDNA3.1 with HA-K48 only ubiquitin, Amp^r^	[59]
pCDNA-HA-Ubi-K27	pCDNA3.1 with HA-K27 only ubiquitin, Amp^r^	[59]
pCDNA-HA	pCDNA3.1 with HA tag, Amp^r^	[59]
pCDNA-HA-*p65*	pCDNA3.1 with HA-*p65*, Amp^r^	This study
pCDNA-HA-*p65*_1-311 aa_	pCDNA3.1 with HA-*p65*_1-311 aa_ (1–311 aa of p65), Amp^r^	This study
pCDNA-HA-*p65*_1-427 aa_	pCDNA3.1 with HA-*p65*_1-427 aa_ (1–427 aa of p65), Amp^r^	This study
pCDNA-HA-*p65*_312-551 aa_	pCDNA3.1 with HA-*p65*_312-551 aa_ (312–551 aa of p65), Amp^r^	This study
pCDNA-HA-*yfp*	pCDNA3.1 with *yfp*-HA, Amp^r^	This study
pCDNA-HA-*traf6 DN*	pCDNA3.1 with HA-*traf6* DN (289–522 aa of TRAF6), Amp^r^	This study
pCDNA-HA-*tak1*	pCDNA3.1 with HA-*tak1*, Amp^r^	This study
pCDNA3.1	CMV promoter, Amp^r^, Neo^r^	Invitrogen
pCDNA-*traf6 DN*	pCDNA3.1 with *traf6 DN* (289–522 aa of TRAF6), Amp^r^	This study
p-*eseJ*_1-242 aa_-3Flag	p-3 × Flag with *eseJ*_1-242 aa_ (1–242 aa of EseJ), Amp^r^	This study
p-*eseJ*_1-532 aa_-3Flag	p-3 × Flag with *eseJ*_1-532 aa_ (1–532 aa of EseJ), Amp^r^	This study
p-*eseJ*_243-532 aa_-3Flag	p-3 × Flag with *eseJ*_243-532 aa_ (243–532 aa of EseJ), Amp^r^	This study
p-*eseJ*_533-1359 aa_-3Flag	p-3 × Flag with *eseJ*_533-1359 aa_ (533–1359 aa of EseJ), Amp^r^	This study
p-NF-κB-Luc	pGL3-basic luciferase reporter vector with NF-κB promoter reporter, Amp^r^	[61]
pRL-TK	Renilla luciferase plasmid, Amp^r^	[61]

Col, colistin; Tet, tetracycline; Amp, ampicillin; Km, kanamycin; r, resistant; s, sensitive; aa, amino acids.

Murine macrophages cell line J774A.1 and human embryonic kidney cell line 293T (HEK 293T) were maintained in Dulbecco’s Modified Eagle Medium (DMEM) (HyClone, Cat No: SH30022.01) supplemented with 10% fetal bovine serum (FBS) (Gibco) at 37°C under a 5% CO_2_ atmosphere.

### Plasmids construction

The DNA fragment encoding p65 was amplified with primers p65-for and p65-rev (Table 2), and inserted into the KpnI and XbaI sites of pCDNA-HA vector by homologous recombination or restriction enzyme digestion-ligation to generate pCDNA-HA-*p65* plasmid. Similarly, pCDNA-HA-*p65*_1–311 aa_, pCDNA-HA-*p65*_1–427 aa_, pCDNA-HA-*p65*_312–551 aa_, pCDNA-*traf6* DN, pCDNA-HA-*traf6* DN, pCDNA-HA-*tak1*, and pCDNA-HA-*yfp* were constructed identically. The *eseJ* gene was amplified with primers EseJ-3Flag-for and EseJ-3Flag-rev (Table 2), digested with BamHI and EcoRI, and ligated to construct p-*eseJ*-3Flag plasmid. Similarly, p-*eseJ*_1-242 aa_-3Flag, p-*eseJ*_1-532 aa_-3Flag, p-*eseJ*_243-532 aa_-3Flag, and p-*eseJ*_533-1359 aa_-3Flag plasmids were generated.

**Table 2:** Oligonucleotides used in this study.

Designation	Nucleotide Sequence
RT-*Il-1β*-m-for	TCAAATCTCGCAGCAGCACATC
RT-*Il-1β*-m-rev	CCAGCAGGTTATCATCATCATCCC
RT-*Il-6*-m-for	AGTTGCCTTCTTGGGACTGA
RT-*Il-6*-m-rev	CCTCCGACTTGTGAAGTGGT
RT-*gapdh*-m-for	TGCACCACCAACTGCTTAGC
RT-*gapdh*-m-rev	GGAAGGCCATGCCAGTGA
RT-*actin*-m-for	CATCCGTAAAGACCTCTATGCCAAC
RT-*actin*-m-rev	ATGGAGCCACCGATCCACA
RT-*rpl13a*-m-for	GAAGCAGATCTTGAGGTTACGGA
RT-*rpl13a*-m-rev	GCAGGCATGAGGCAAACAGT
RT-p65-m-for	TGCGATTCCGCTATAAATGCG
RT-p65-m-rev	ACAAGTTCATGTGGATGAGGC
EseG-pSUB11-for	GAAAACCCGCGCGTTTCTCATTCAAACTCTCCGCCAAAACGGCGTCGAGTCTGACTACAAAGACCATGACGGT
EseG-pSUB11-Flag-rev	CTGTGTATCGGGTGCATACCTGAATTGATGTGCGAGCGGCGCGCGGGCATCATATGAATATCCTCCTTAGT
EseJ-pSUB11-Flag-for	AGCACAGCCCGCTGAACGGGCATGAGACGGCGGCGGCGATAGACTACAAAGACCATGACGGTGATTATAAAGATCATGACATCGACTACAAG
EseJ-pSUB11-Flag-rev	TAAGTCACGGTGCTACGCCGCCTGACGCGGCGGCGTAAATCCCATATGAATATCCTCCTTAGT
EseJ-3Flag-for	CATCGATAGATCTGATATCGGTACCATGGTGAATGCTTTTACGTTATCCCCCGCGC
EseJ_243_-3Flag-for	CATCGATAGATCTGATATCGGTACCATGCCGCGCTATACGTTCAATCTCCTGTTCC
EseJ_1-242_–3Flag-rev	TGTAGTCAGCCCGGGATCCTCTAGACTGCAGCAGAGAGCCGATC
EseJ_1-532_–3Flag-rev	TGTAGTCAGCCCGGGATCCTCTAGACAAGGCGCTGAAGTAGTCC
EseJ_533-1359_-for	CGATAGATCTGATATCGGTACCATGAAGCAGCGCAGCCAGGCGCT
EseJ_533-1359_-rev	TTTGTAGTCAGCCCGGGATCCTCTAGATATCGCCGCCGCCGTCT
EseJ-3Flag-rev	TTTGTAGTCAGCCCGGGATCCTCTAGATATCGCCGCCGCCGTCT
p65-for	ATATCAAGCTTAGCGCTGGTACCATGGACGAACTGTTCCCCC
p65_311_-rev	CGGGTTTAAACGGGCCCTCTAGATTAGCTCTTGAAGGTCTCATATGTCCTTTTACG
p65_427_-rev	CGGGTTTAAACGGGCCCTCTAGATTACTTGGGGGCAGGTGGGGCC
p65_312_-for	ATATCAAGCTTAGCGCTGGTACCATGATCATGAAGAAGAGTCCTTTC
p65-rev	CGGGTTTAAACGGGCCCTCTAGATTAGGAGCTGATCTGACTCAGC
YFP-for	CGGGATCCATGGTGAGCAAGGGCGAG
YFP-HA-rev	GGAATTCTTAAGCGTAATCTGGAACATCGTATGGGTACTTGTACAGCTCGTCCATGCC
TAK1-for	GGGGTACCATGTCGACAGCCTCCGCC
TAK1-rev	GCTCTAGATCATGAAGTGCCTTGTCGTTTCTGCTGTTGGC
TRAF6 DN-for	GATATCAAGCTTAGCGCTGGTACCATCTCAGAGGTCCGGAATTT
TRAF6 DN-rev	CGGGTTTAAACGGGCCCTCTAGACTATACCCCTGCATCAGTA
RT-*Il-1β*-z-for	GACTTCGCAGCACAAAATGA
RT-*Il-1β*-z-rev	ATACGCGGTGCTGATAAACC
RT-*Il-6*-z-for	GTCCCCGTGTTCAGCAGTAT
RT-*Il-6*-z-rev	TCATCACGCTGGAGAAGTTG
RT-*Tnf-α*-z-for	TACCTGAGCCACACCATCAA
RT-*Tnf-α*-z-rev	ACTCTCACTGCATCGGCTTT
RT-*gapdh*-z-for	TCACATTAAGGGTGGTGCAA
RT-*gapdh*-z-rev	AACCTGGTGCTCCGTGTATC
RT-*actin*-z-for	CAAGGAAGTCAGCGCATACA
RT-*actin*-z-rev	GAGACTCGTGGTGCATCTCA

### Generation of Flag-tagged *eseG* and *eseJ* strains via chromosomal epitope tagging

Chromosomal *eseJ* or *eseG* gene was C-terminally tagged with Flag using λ Red recombination [62,63]. A Flag-encoding sequence followed by a kanamycin resistance selection marker was amplified using plasmid pSUB11 as the template. For each target gene, a pair of chimeric primers was designed with three functional modules arranged in the 5’ to 3’ orientation (Table 2): (1) a 30-nucleotide (nt) homology to the 3’-end of *eseJ* or *eseG* gene, with the endogenous stop codon omitted to ensure in-frame translational fusion of the Flag-tag to the C-terminus of the target protein; followed by (2) the Flag-encoding sequence; and (3) an additional 30-nt homology to the chromosomal region immediately downstream of *eseJ* or *eseG* gene. The resulting PCR products were electroporated into Red recombinase-expressing *E. piscicida* wild type strains harboring pKD46. Transformants were selected on tryptic soy agar (TSA) containing 50 µg/mL kanamycin and 12.5 µg/mL colistin. Next, the temperature-sensitive pKD46 was cured by culturing the obtained colony at 37 °C, after which it was screened negatively on TSA containing kanamycin. The acquired strains were then verified by immunoblotting using an anti-Flag antibody (M2, Sigma).

### Infection of J774A.1 macrophages

J774A.1 macrophages were seeded and infected as described [64] with slight modification. Briefly, J774A.1 macrophages were seeded at 5 × 10^5^ cells per well, infected with *E. piscicida* opsonized with naïve mouse serum at a multiplicity of infection (MOI) of 20, centrifuged at 170 × g for 5 min at room temperature and incubated at 35°C. At time zero post infection, monolayers were rinsed twice and incubated in DMEM with 50 μg/mL gentamicin. At 2 h or 3 h post infection, supernatants and adherent cells were collected for immunoblot, immunofluorescence microscopy, and RNA extraction, respectively; at 5 h post infection, supernatants were analyzed by Luminex bead-based multiplex immunoassay (Wayen Biotechnologies, Shanghai).

To inhibit TAK1, J774A.1 macrophages were pretreated with 50 μM TAKinib (Selleck) in 10% FBS-DMEM for 1 h prior to infection, and this treatment was continued throughout the infection. Supernatants and cell lysates were collected at 3 h post infection. To inhibit proteasomal degradation, J774A.1 macrophages were treated with 20.0 μM MG132 (a proteasome inhibitor) for 3 h prior to sampling. Similarly, 293T cells were treated with the inhibitor for 8 h before sampling. To inhibit autophagy, J774A.1 macrophages were treated with 5 mM 3-methyladenine (3-MA, an autophagy inhibitor) for 3 h before sampling. To inhibit NF-κB, the infected J774A.1 cells were treated with 2.0 μM carpaine (an NF-κB inhibitor) for 6 h prior to and throughout the 3 h infection period.

### Immunoprecipitation

J774A.1 macrophages were seeded at 10^7^ cells per 10 cm dish, *E. piscicida* strains opsonized with naïve mouse serum (30 min, 35°C) were infected at a MOI of 20, and then incubated at 35°C for 1 h. The monolayers were rinsed twice and incubated in 4 mL DMEM supplemented with 50 μg/mL gentamycin and 20 μM MG132 for 3 h. Cells were then harvested and lysed with IP lysis buffer (25 mM Tris-HCl pH 7.4, 150 mM NaCl, 1 mM EDTA, 1% NP-40, 5% glycerol and protease inhibitor cocktail) for immunoprecipitation.

HEK293T cells were seeded at 5 × 10⁶ cells per 10 cm culture dish, cultured 18 h prior to transfection with NEOfect transfection reagent using plasmids: p-3Flag, p-*yfp*-3Flag, p-*eseQ*-3Flag, p-*eseJ*-*3*Flag, p-*eseJ*_1-242 aa_-*3*Flag, p-*eseJ*_243-532 aa_-*3*Flag, p-*eseJ*_533-1359 aa_-*3*Flag, pCDNA-HA-*tak1*, pCDNA-HA-*p65*, pCDNA-HA-*p65*_1–311 aa_, pCDNA-HA-*p65*_1–427 aa_, pCDNA-HA-*p65*_312–551 aa_, pCDNA-HA-*traf6* DN, pcDNA-HA-Ubi-WT/K63/K48/K27. Transfected cells were incubated at 37°C under 5% CO_2_ for 24 h, then pretreated with 20 μM MG132 for 6 h before lysis in IP lysis buffer for 30 min on ice. Cell lysates were incubated with 2 μg primary antibody: anti-HA antibody (HuaBio), anti-Flag antibody (HuaBio), anti-TAK1 antibody (CST), or anti-p65 antibody (CST) at 4°C for 4 h, followed by protein G agarose beads added, and incubated overnight at 4°C with rotation. The agarose beads were pelleted, washed seven times with ice-cold IP lysis buffer and immunoprecipitates eluted with sample buffer for immunoblot analysis.

### Immunoblotting assay

Proteins from infected J774A.1 macrophages supernatants and cell lysates were precipitated using methanol-chloroform method as described previously [65]. Pellets were air-dried, dissolved in 1.5 × SDS loading buffer, and resolved alongside HEK293T lysates and yellow catfish (*Pelteobagrus fulvidraco*) leukocyte samples on NuPAGE 10% or 12% Bis-Tris gels (Novex). Proteins were transferred to 0.2-μm PVDF membranes, blocked with 5% skim milk, and incubated overnight at 4 °C with primary antibodies: rabbit anti-TAK1 (CST), rabbit anti-phosphorylated TAK1 (CST), rabbit anti-IKKα/β (Beyotime), rabbit anti-phosphorylated IKKα/β (CST), rabbit anti-IκBα (Beyotime), mouse anti-phosphorylated IκBα (CST), rabbit anti-HA (CST), mouse anti-Flag (Sigma), rabbit anti-Flag (HuaBio), rabbit anti-TRAF6 (Beyotime), rabbit anti-p65 (HuaBio), rabbit anti-HA(HuaBio), rabbit anti-K48-ubi (Selleck), rabbit anti-IL-1β (Atagenix) monoclonal antibody at a 1:1000 dilution, mouse anti-p65 (CST) and rabbit anti-phosphorylated p65 monoclonal antibody (Ser 536, CST) at a 1:2000 dilution, rabbit anti-Actin polyclonal antibody (ABclonal), anti-GAPDH polyclonal antibody (Sangon) at a 1:5,000 dilution. Membranes were probed with HRP-conjugated goat anti-rabbit or anti-mouse IgG (1:5000; Millipore) for detection.

For protein stability analysis, HEK293T cells co-transfected with pCDNA-HA-*p65* and either p-*eseJ*-3Flag or p-*yfp*-3Flag were treated with 50 μM cycloheximide (CHX, a protein synthesis inhibitor) at 18 h post-transfection. Cells were harvested after 0 h, 4 h, 8 h, or 12 h of treatment, lysed in IP lysis buffer, and analyzed for HA-p65 protein levels by immunoblotting using GAPDH as the loading control.

### RNA extraction and RT-PCR

Total RNA was isolated from J774A.1 macrophages infected with *E. piscicida* Δ*fimH* and Δ*eseJ*Δ*fimH* strains for 3 h, and from zebrafish larvae infected with corresponding strains for 24 h and 48 h, using TRIzol reagent (Invitrogen; 1 mL per sample). Complementary cDNA was synthesized using the RevertAid First Strand cDNA Synthesis kit (Thermo).

Quantitative real-time PCR (qRT-PCR) was performed using SYBR green reagent on a CFX96 Real-Time PCR System (Bio-Rad). Gene expression levels were normalized to reference genes and relative transcript levels were calculated using the comparative ^ΔΔ^CT method. For J774A.1 macrophages, *p65* expression was normalized to *gapdh* and *β-actin*, *Il-1β* and *Il-6* expression were normalized to *gapdh* and *rpl13a*. For zebrafish larvae, *Il-1β, Il-6, and Tnf-α* expression were normalized to *gapdh* and *β- actin* (Table 2).

### Immunofluorescence microscopy

J774A.1 macrophages on coverslips were infected with Δ*fimA*[GFP] or Δ*eseJ*Δ*fimA*[GFP] *E. piscicida* strains at an MOI of 20. At 3 h post infection, cells were washed three times with PBS and fixed in 4% paraformaldehyde (PFA) overnight at 4°C. Fixed cells were permeabilized, blocked, and incubated with rabbit anti-p65 primary antibody (HuaBio), followed by staining with Alexa Fluor 594-conjugated secondary antibody (red). Bacterial GFP expression provided green fluorescence, and nuclei were counterstained with DAPI (blue). Images were acquired on a confocal laser-scanning microscope (LSM 710; Carl Zeiss). Nuclear translocation of p65 in infected cells (GFP-positive) was quantified by counting cells with predominant nuclear p65 signal relative to DAPI localization. Data represent ≥12 fields per coverslip from three independent coverslips per infection condition.

### NF-κB luciferase reporter assay

HEK293T cells were co-transfected with p-*eseJ*-3Flag plasmid or empty p-3Flag vector control, along with NF-κB luciferase reporter gene plasmid (p-NF-κB-Luc) and pRL-TK Renilla luciferase plasmid for 16 h before cells were treated with 2.0 μM carpaine (an NF-κB inhibitor) and 15.0 ng/ml TNF-α for 8 h before harvest. In parallel, HEK293T cells were co-transfected with p-*eseJ*-3Flag plasmid or empty p-3Flag vector with or without pCDNA-HA-*traf6 DN* plasmid, in combination with p-NF-κB-Luc and pRL-TK plasmids. Cells were pretreated with 15 ng/ml TNF-α for 8 h before harvest. At 24 h post-transfection, cells were lysed for reporter luciferase activity using the Dual-Luciferase reporter assay system (Vazyme). All the luciferase activity values were achieved against the Renilla luciferase control. Firefly luciferase activity was normalized to the corresponding Renilla luciferase activity for each sample. Transfection of each construct was performed in triplicate in each assay, and the average value of triplicate replicates was calculated for subsequent analysis.

### Zebrafish infection

Zebrafish larvae at 4 days post fertilization were subjected to immersion infection with *E. piscicida* (5 × 10^8^ CFU/mL) at 25°C for 6 h. Infected larvae (n = 30/group) were transferred to aseptic culture water in petri dishes. At 12 h, 24 h, or 36 h post infection, infected zebrafish larvae were collected for RNA extraction.

### Isolating yellow catfish leukocytes

Leukocytes were isolated from head kidneys of healthy yellow catfish (*Pelteobagrus fulvidraco*). Tissues were minced in DMEM supplemented with 100 U/mL penicillin, 100 μg/mL streptomycin and 20 U/mL sodium heparin, then filtered through 100-μm sterile cell strainer. Single-cell suspension was gently layered onto discontinuous 34%/51% Percoll gradients and centrifuged at 400 × g for 40 min at 4 °C. Interface leukocytes were collected carefully, washed three times in DMEM containing 2% FBS, and resuspended in DMEM containing 10% FBS. The isolated leukocytes were seeded into 24-wells plate at a density of 2.5 × 10^6^ cells per well, incubated for 2 h at 28°C, and infected with *E. piscicida* at MOI of 20 for 5 h. After infection, the cell lysates and supernatants were collected and precipitated for immunoblotting.

### Statistical analysis

All experiments were repeated at least three times independently and were presented as mean ± SD (standard deviation) or as mean ± SEM (standard error of mean). Probability (*P*) values were calculated by using two-way analysis of variance and least-significant-difference (LSD) or Student’s *t*-test. A *P* value of < 0.05 was considered significantly different.

## Supporting information

S1 Table Homologies of *E. piscicida* EseJ with proteins in diverse Gram-negative pathogens. (DOCX)

S1 Raw image Raw image. (PDF)
