# Supplementary material for: The Edwardsiella T3SS effector EseJ enhances NF-κB signaling by stabilizing p65 and promoting TAK1 phosphorylation
Source: PLoS Pathog. 2026 Sep 15;22(9):e1014260. doi: 10.1371/journal.ppat.1014260 (PMC13577494; doi:10.1371/journal.ppat.1014260)
Supplement: Supplementary file 1 [file ppat.1014260.s002.pdf]

Fig 1A

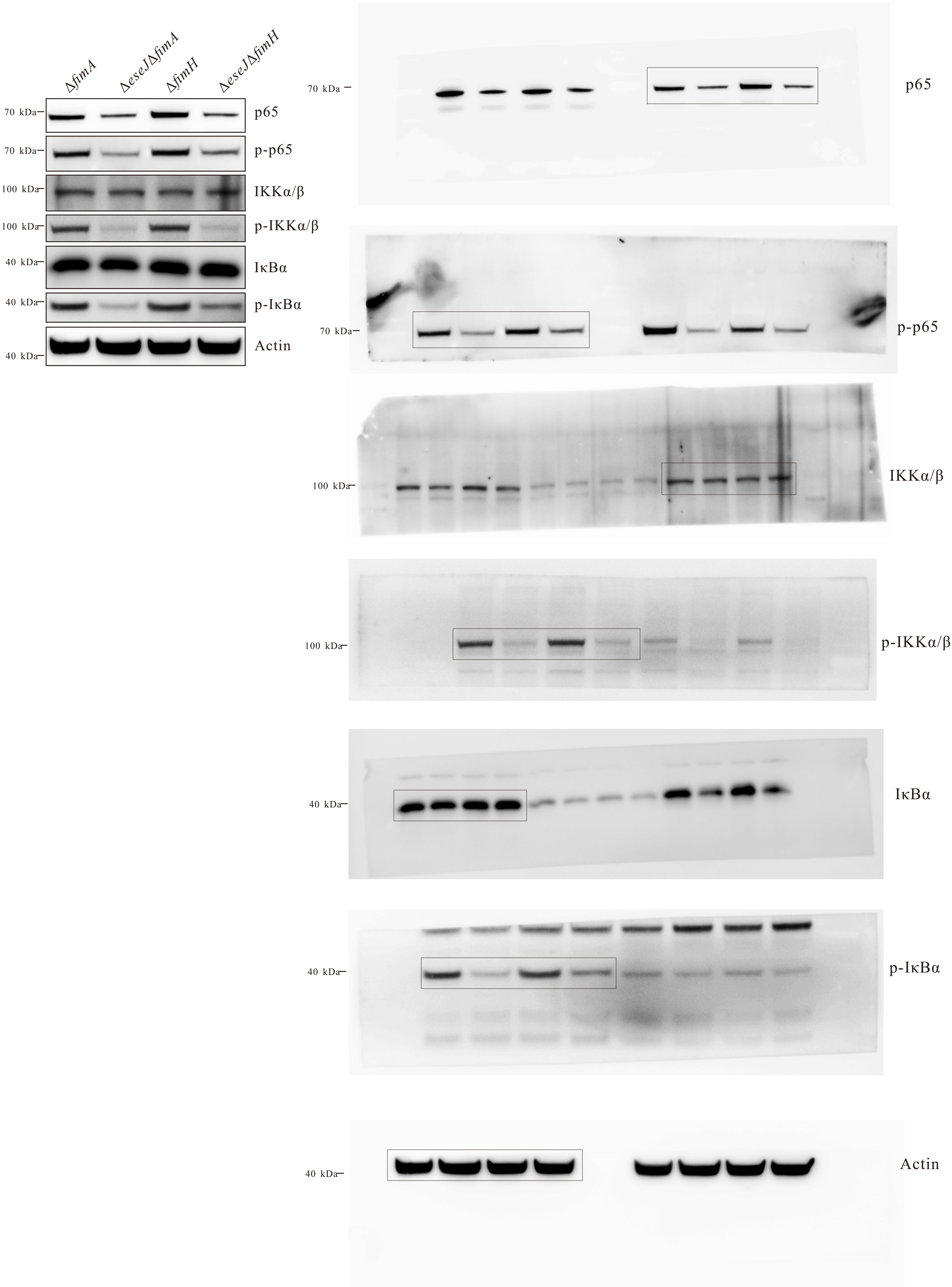

ΔfimA
ΔeseJΔfimA
ΔfimH
ΔeseJΔfimH
70 kDa
p65
p-p65
100 kDa
IKKα/β
p-IKKα/β
40 kDa
IκBα
p-IκBα
Actin

Fig 2A

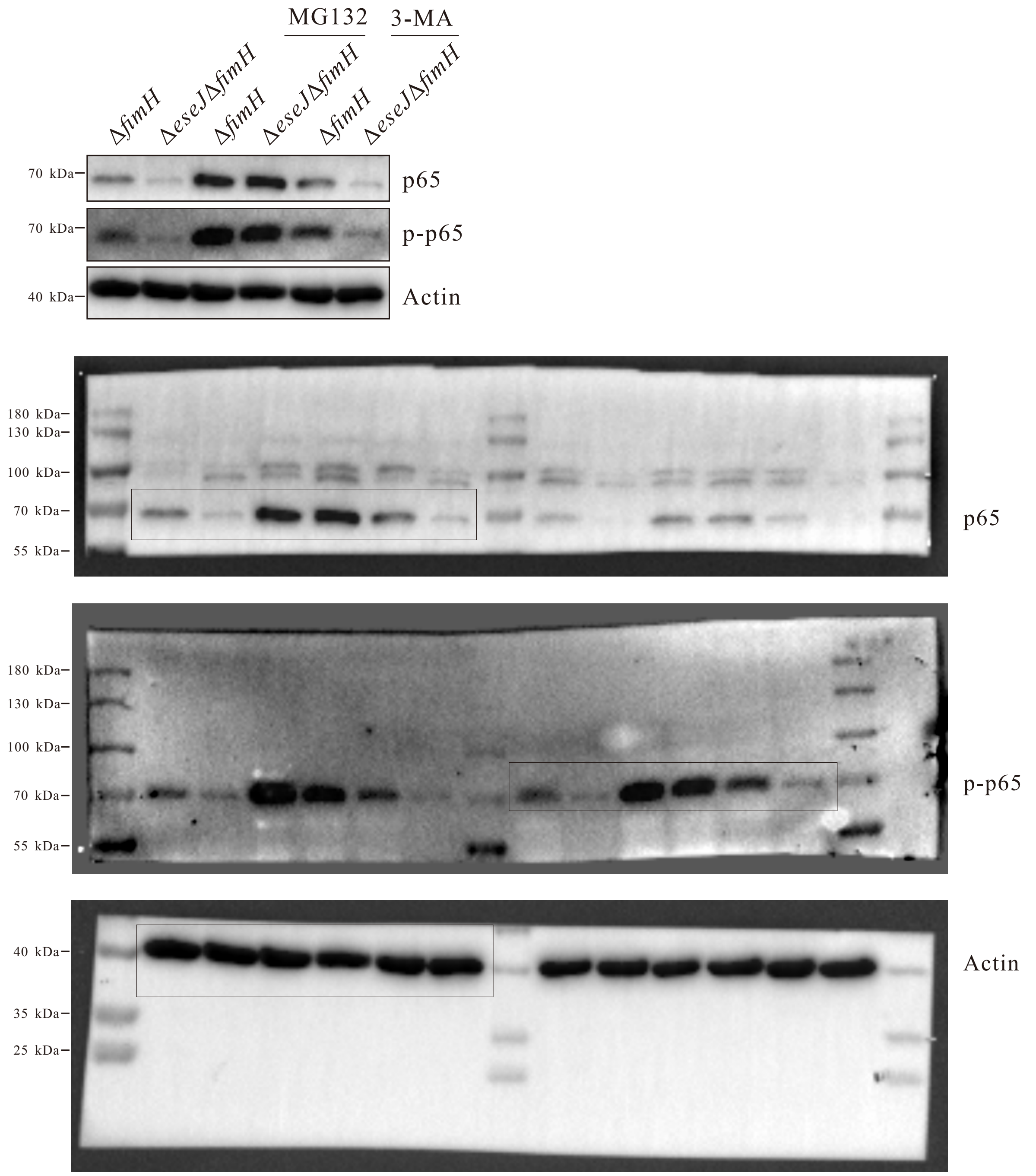

MG132
3-MA
ΔfimH
ΔeseJΔfimH
ΔfimH
ΔeseJΔfimH
ΔfimH
ΔeseJΔfimH
70 kDa
p65
70 kDa
p-p65
40 kDa
Actin
180 kDa
130 kDa
100 kDa
70 kDa
55 kDa
p65
180 kDa
130 kDa
100 kDa
70 kDa
55 kDa
p-p65
40 kDa
35 kDa
25 kDa
Actin

Fig 2C

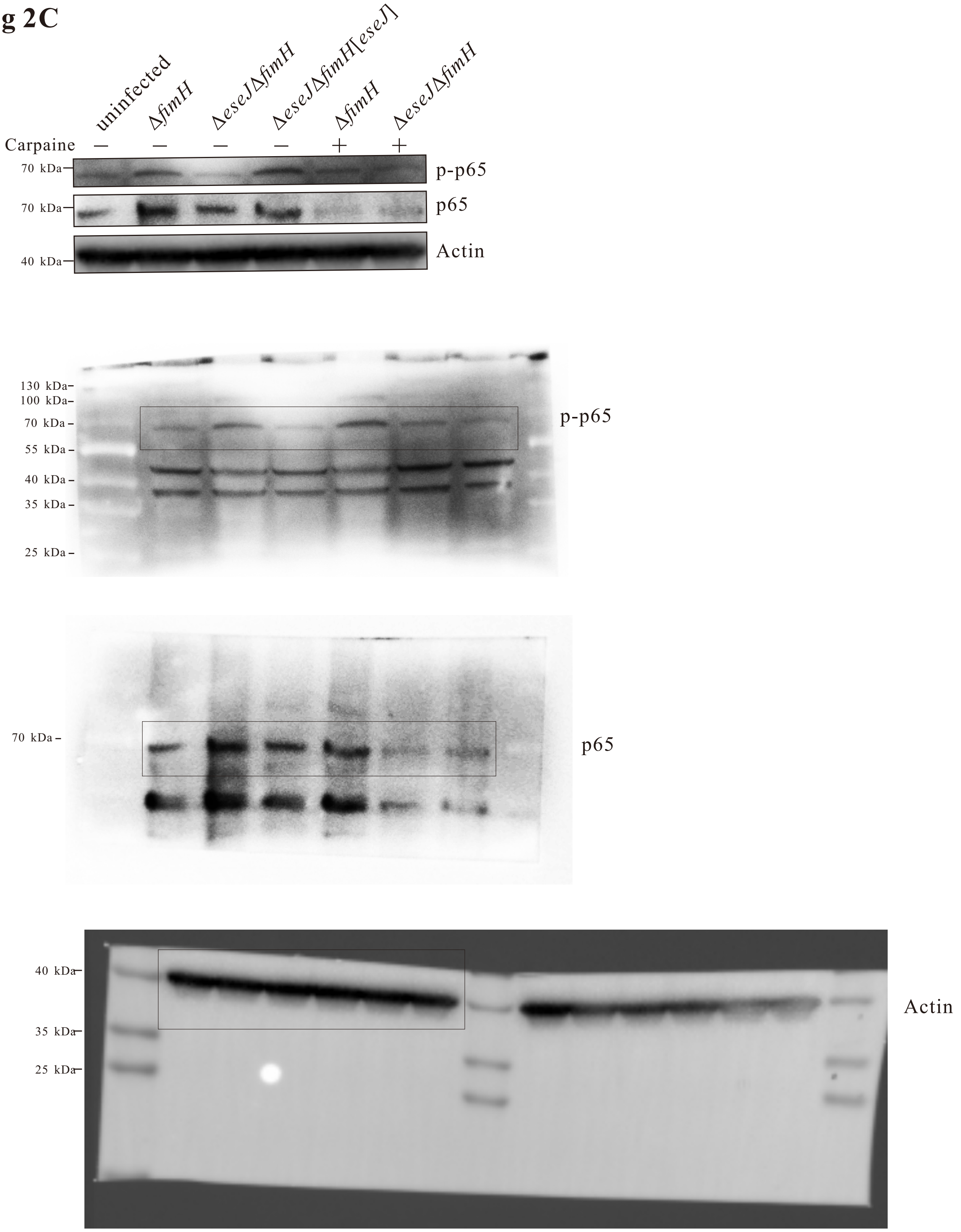
uninfected
ΔfimH
ΔeseJΔfimH
ΔeseJΔfimH[eseJ]
ΔfimH
ΔeseJΔfimH
Carpaine − − − − + +
70 kDa
p-p65
70 kDa
p65
40 kDa
Actin
130 kDa
100 kDa
70 kDa
55 kDa
40 kDa
35 kDa
25 kDa
p-p65
70 kDa
p65
40 kDa
35 kDa
25 kDa
Actin

Fig 2E

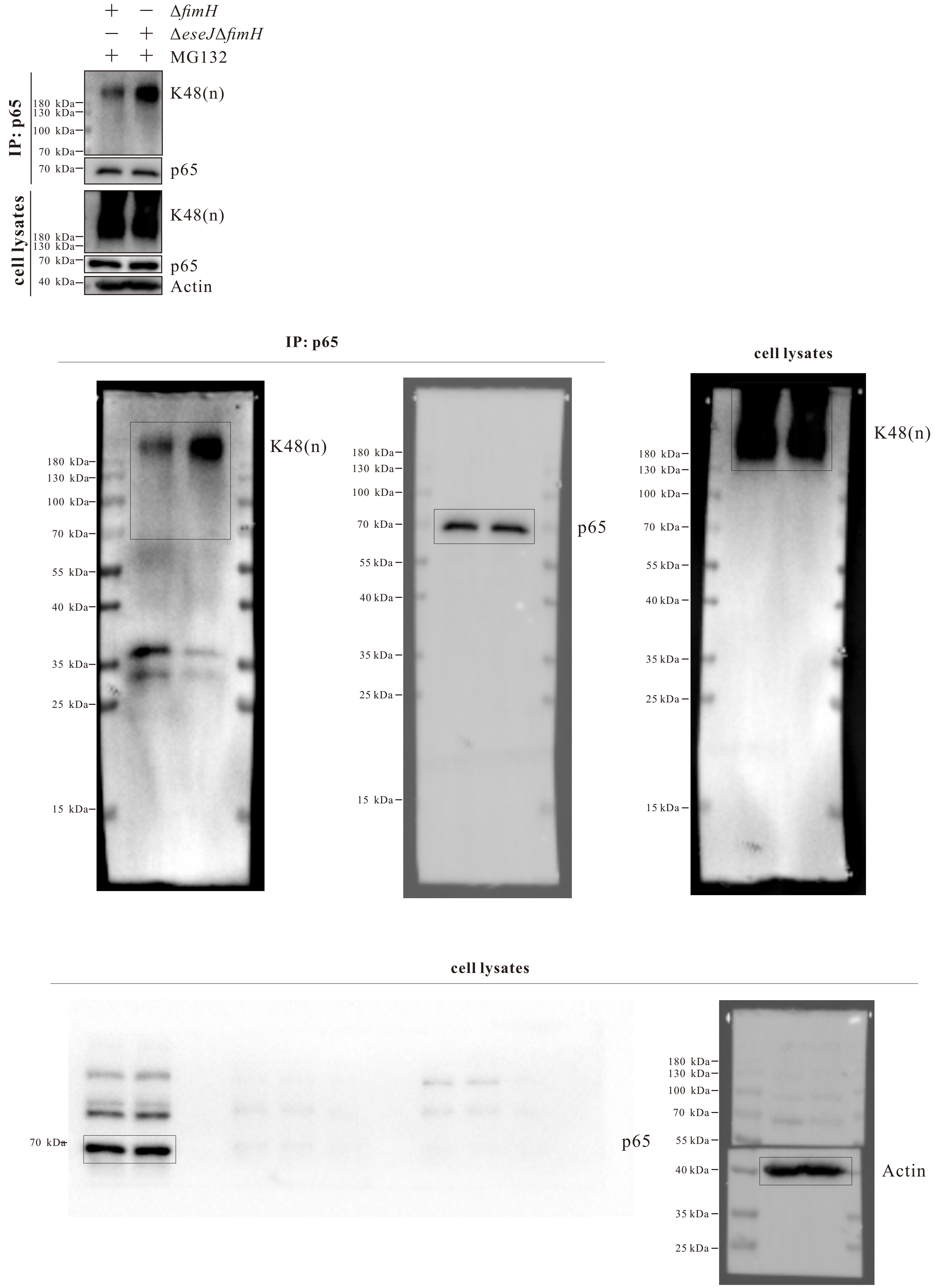

+ − ΔfimH
− + ΔeseJΔfimH
+ + MG132
IP: p65
cell lysates
K48(n)
p65
Actin
180 kDa
130 kDa
100 kDa
70 kDa
55 kDa
40 kDa
35 kDa
25 kDa
15 kDa

Fig 3A

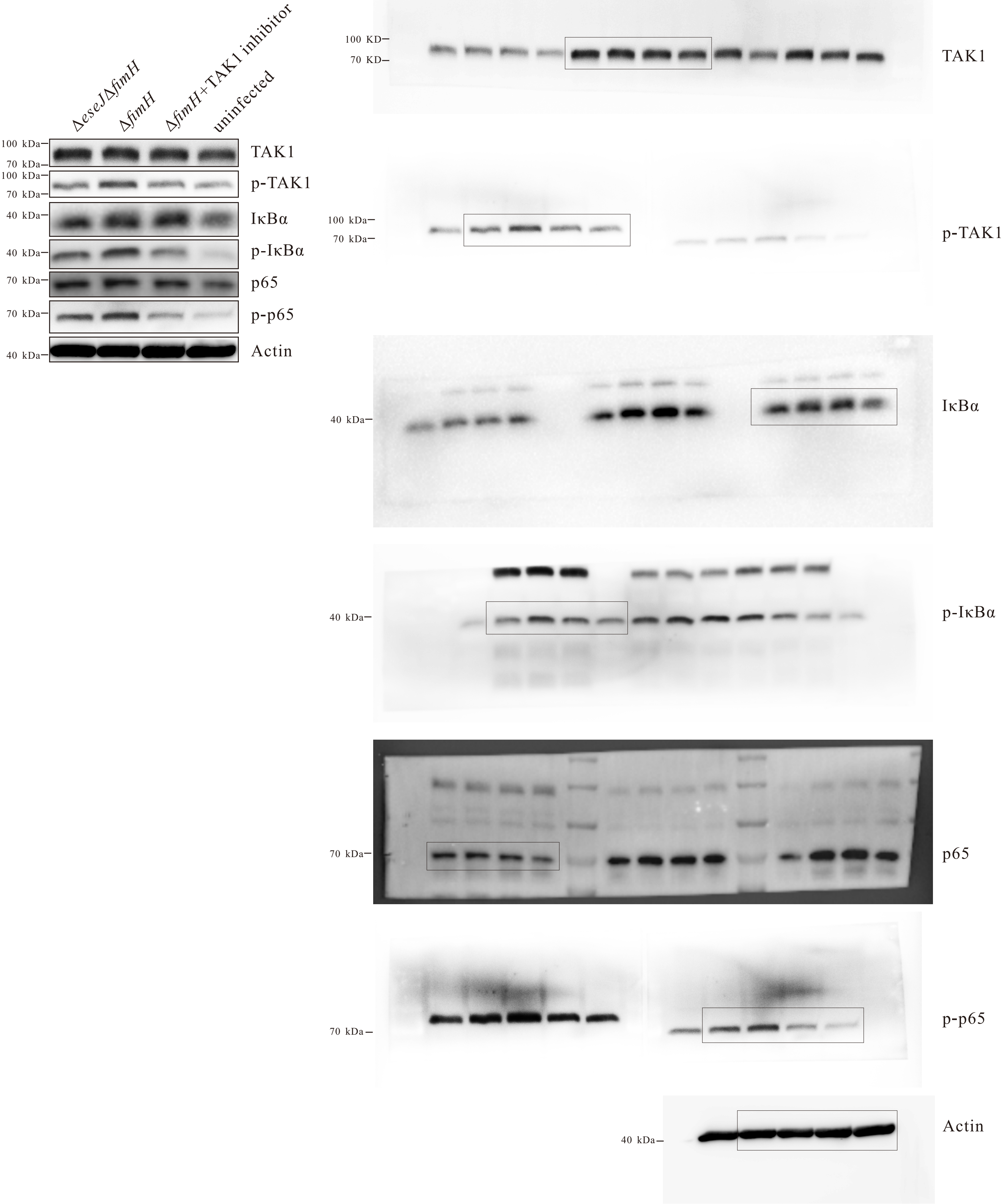
ΔeseJΔfimH
ΔfimH
ΔfimH+TAK1 inhibitor
uninfected
100 kDa
70 kDa
40 kDa
100 KD
70 KD
TAK1
p-TAK1
IκBα
p-IκBα
p65
p-p65
Actin

Fig 4A

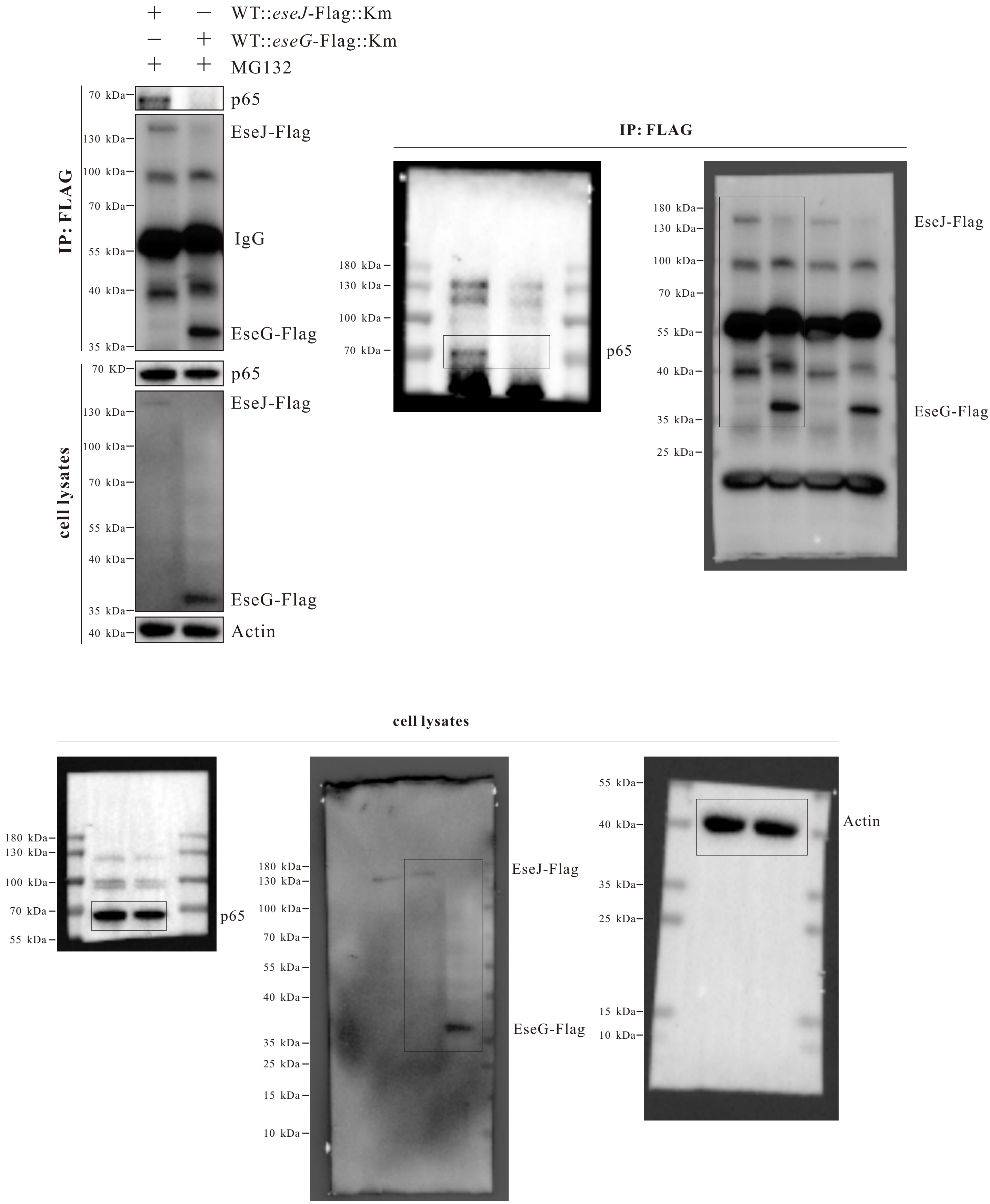

+ − WT::eseJ-Flag::Km
− + WT::eseG-Flag::Km
+ + MG132
IP: FLAG
cell lysates
p65
EseJ-Flag
IgG
EseG-Flag
Actin
70 kDa
130 kDa
100 kDa
55 kDa
40 kDa
35 kDa
70 KD
180 kDa
25 kDa
15 kDa
10 kDa

Fig 4B

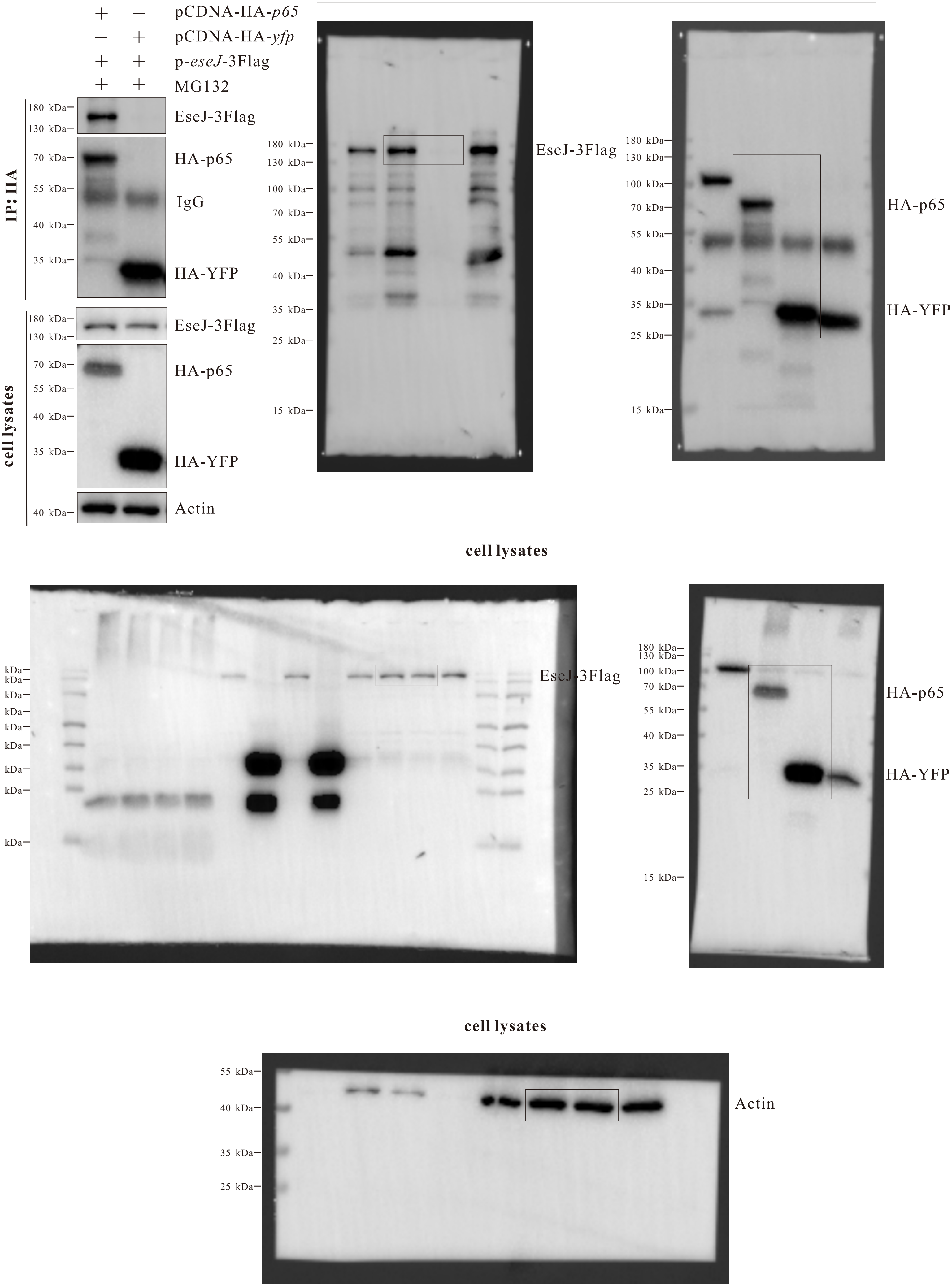

pCDNA-HA-p65
pCDNA-HA-yfp
p-eseJ-3Flag
MG132
IP: HA
cell lysates
EseJ-3Flag
HA-p65
IgG
HA-YFP
Actin
180 kDa
130 kDa
100 kDa
70 kDa
55 kDa
40 kDa
35 kDa
25 kDa
15 kDa

Fig 4C

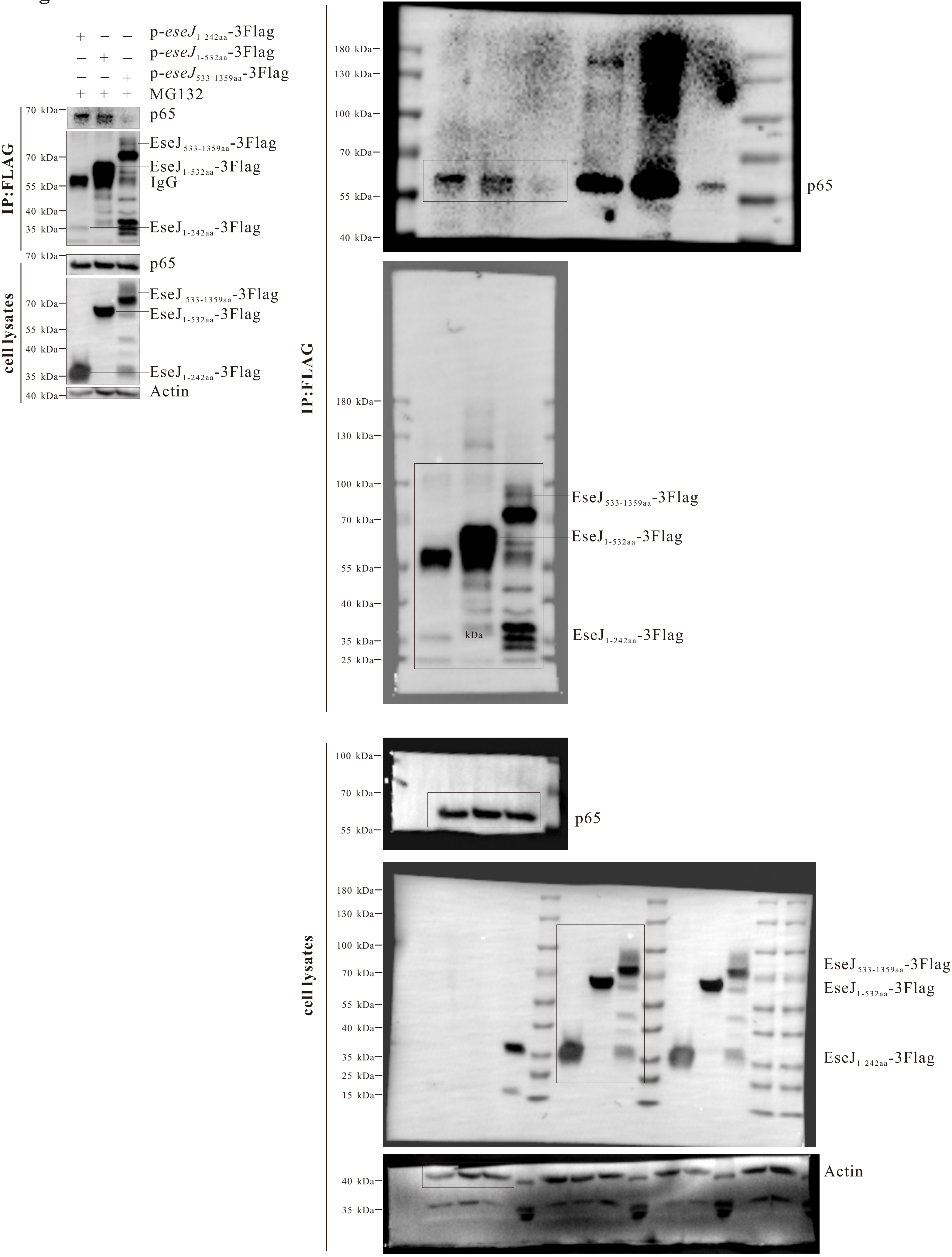
p-eseJ1-242aa-3Flag
p-eseJ1-532aa-3Flag
p-eseJ533-1359aa-3Flag
MG132
IP:FLAG
cell lysates
p65
EseJ533-1359aa-3Flag
EseJ1-532aa-3Flag
IgG
EseJ1-242aa-3Flag
Actin
180 kDa
130 kDa
100 kDa
70 kDa
55 kDa
40 kDa
35 kDa
25 kDa
15 kDa

Fig 4D

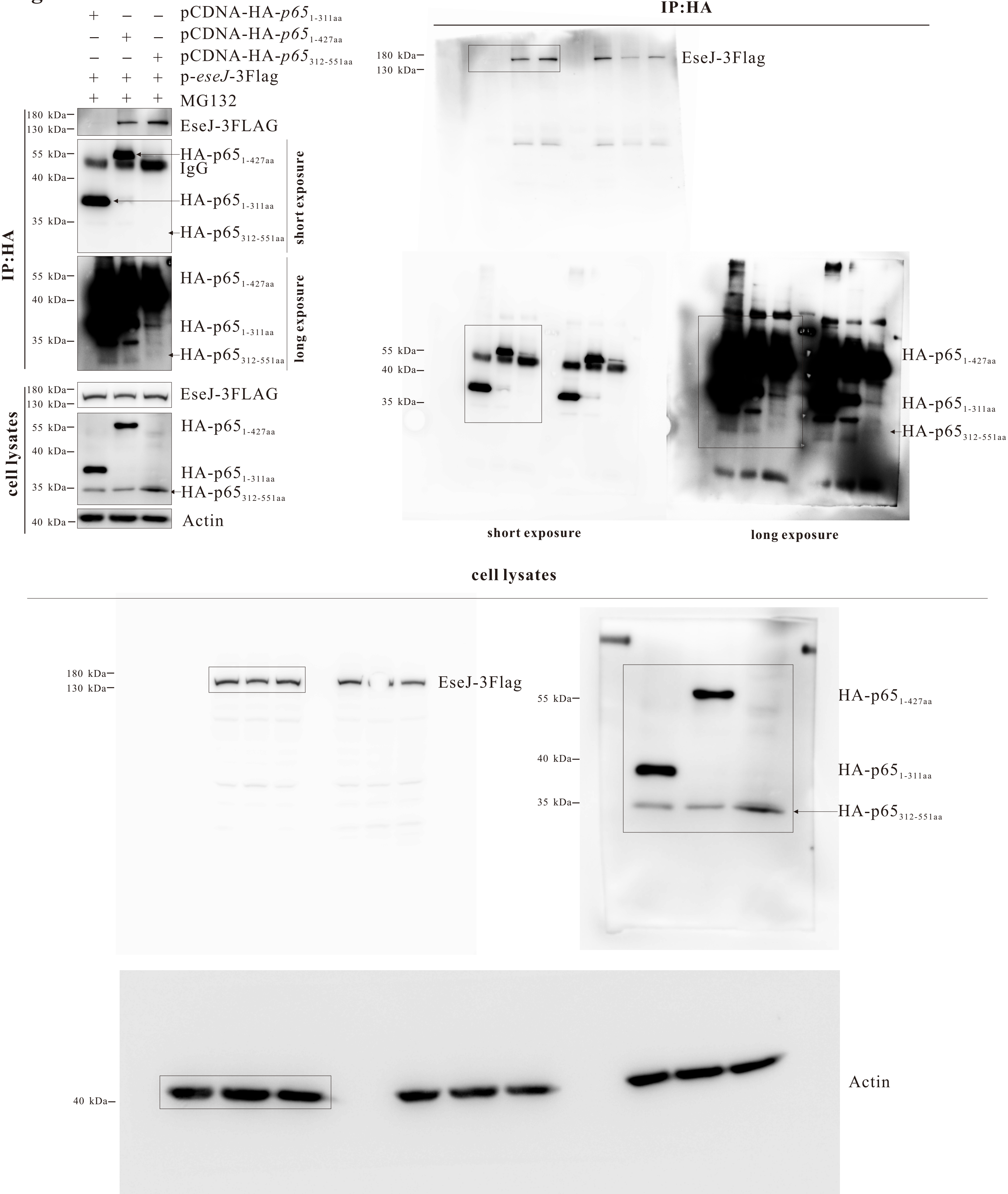

pCDNA-HA-p65 1-311aa
pCDNA-HA-p65 1-427aa
pCDNA-HA-p65 312-551aa
p-eseJ-3Flag
MG132
180 kDa
130 kDa
55 kDa
40 kDa
35 kDa
EseJ-3FLAG
HA-p65 1-427aa
IgG
HA-p65 1-311aa
HA-p65 312-551aa
short exposure
long exposure
IP:HA
cell lysates
Actin
EseJ-3Flag

Fig 4E

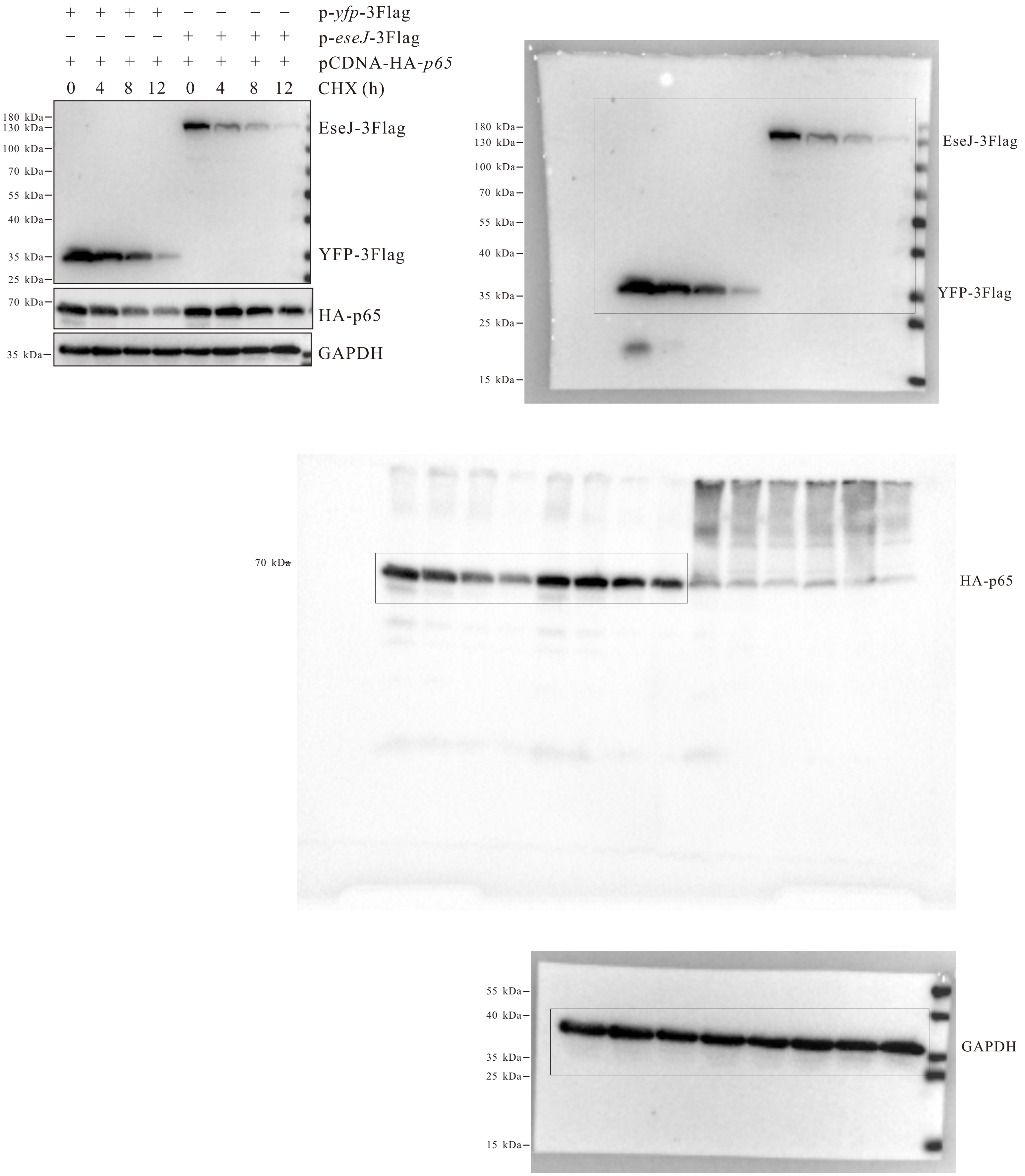
p-yfp-3Flag
p-eseJ-3Flag
pCDNA-HA-p65
CHX (h)
0 4 8 12 0 4 8 12
180 kDa
130 kDa
100 kDa
70 kDa
55 kDa
40 kDa
35 kDa
25 kDa
15 kDa
EseJ-3Flag
YFP-3Flag
HA-p65
GAPDH

Fig 4G

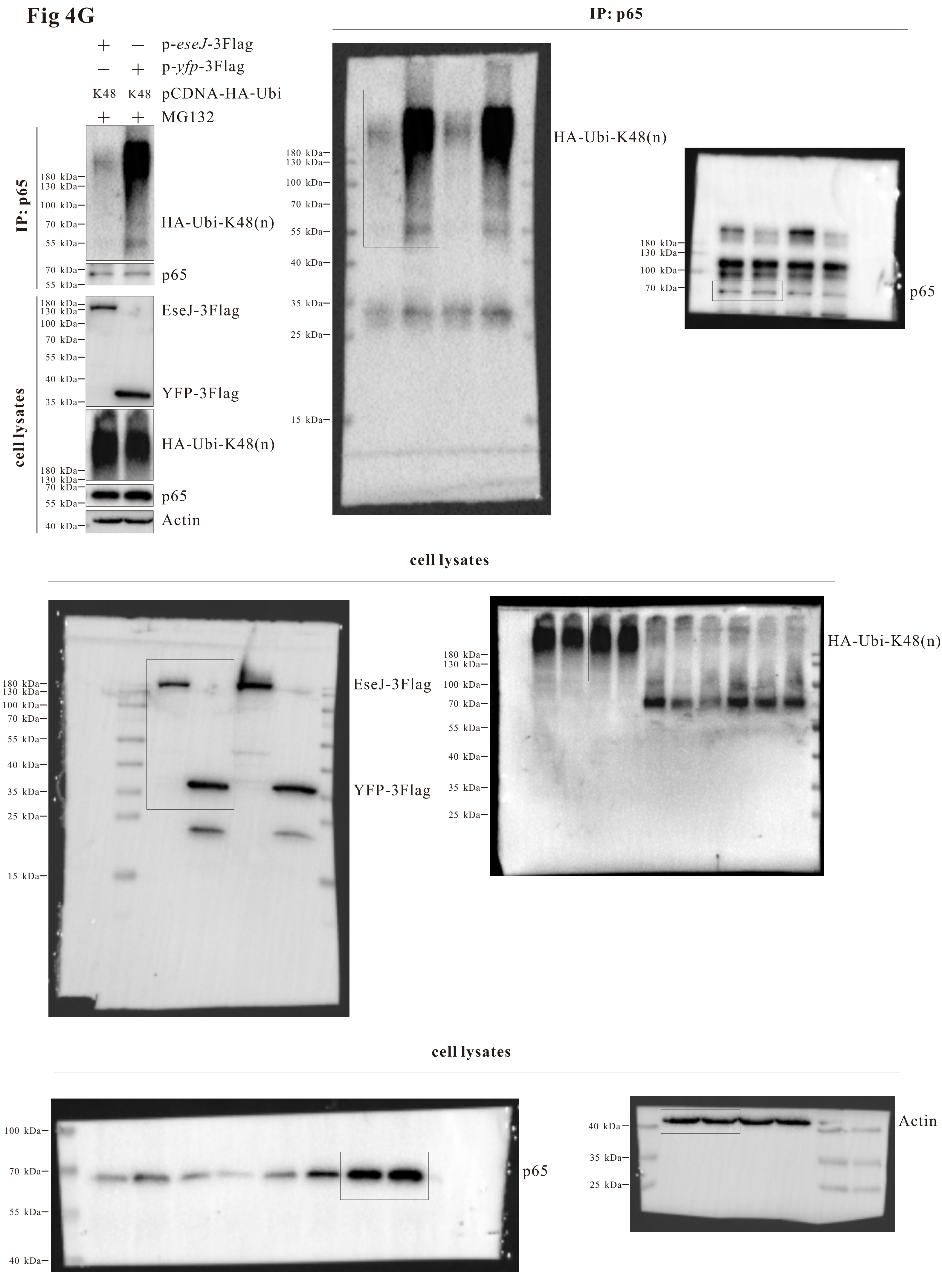

IP: p65
p-eseJ-3Flag
p-yfp-3Flag
pCDNA-HA-Ubi
MG132
K48 K48
IP: p65
cell lysates
HA-Ubi-K48(n)
p65
EseJ-3Flag
YFP-3Flag
HA-Ubi-K48(n)
p65
Actin
180 kDa
130 kDa
100 kDa
70 kDa
55 kDa
40 kDa
35 kDa
25 kDa
15 kDa
cell lysates
EseJ-3Flag
YFP-3Flag
HA-Ubi-K48(n)
cell lysates
p65
Actin

Fig 5A

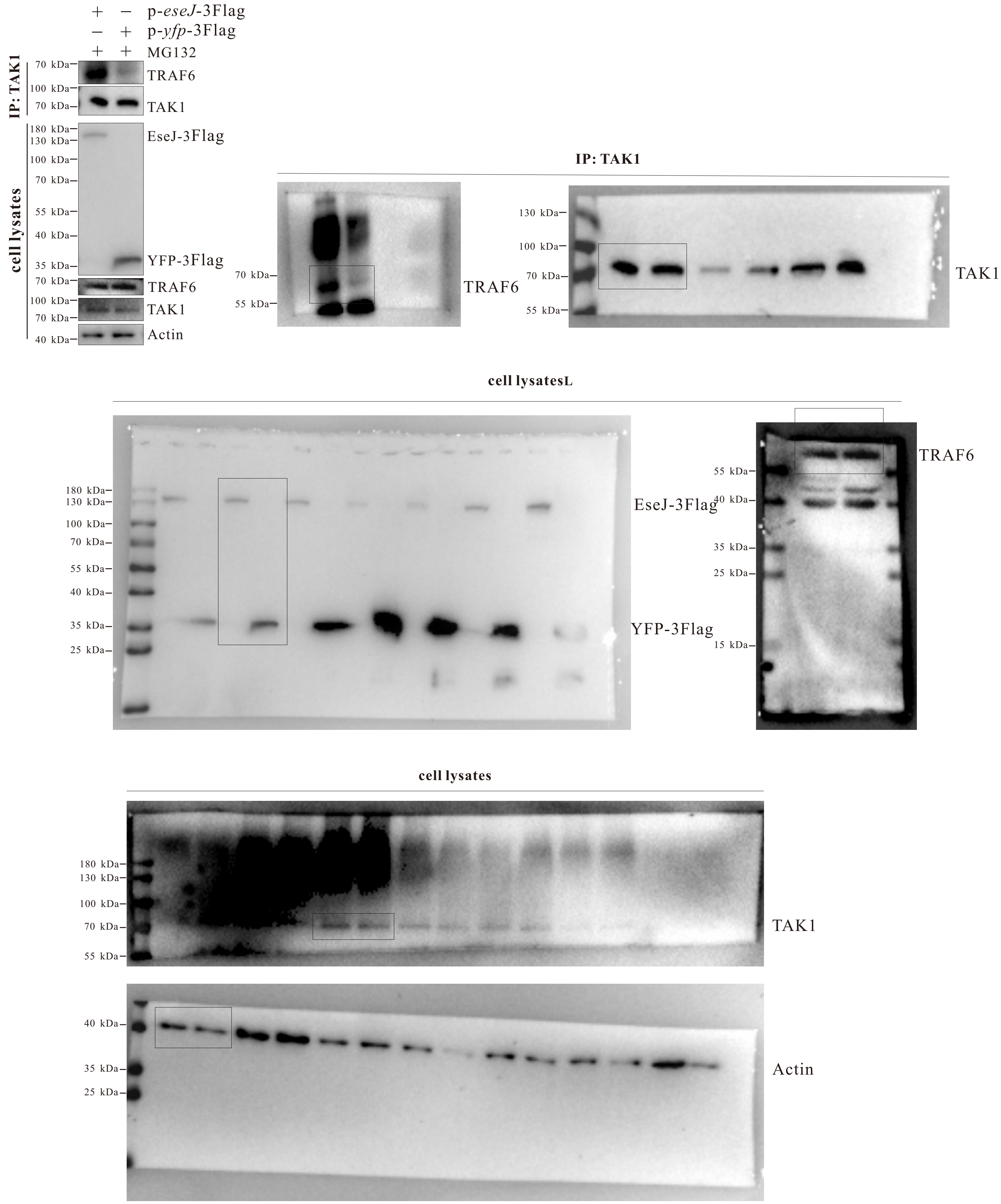

p-*eseJ*-3Flag
p-*yfp*-3Flag
MG132
IP: TAK1
cell lysates
TRAF6
TAK1
EseJ-3Flag
YFP-3Flag
Actin
70 kDa
100 kDa
180 kDa
130 kDa
55 kDa
40 kDa
35 kDa
IP: TAK1
cell lysatesL
25 kDa
15 kDa
cell lysates

Fig 5B

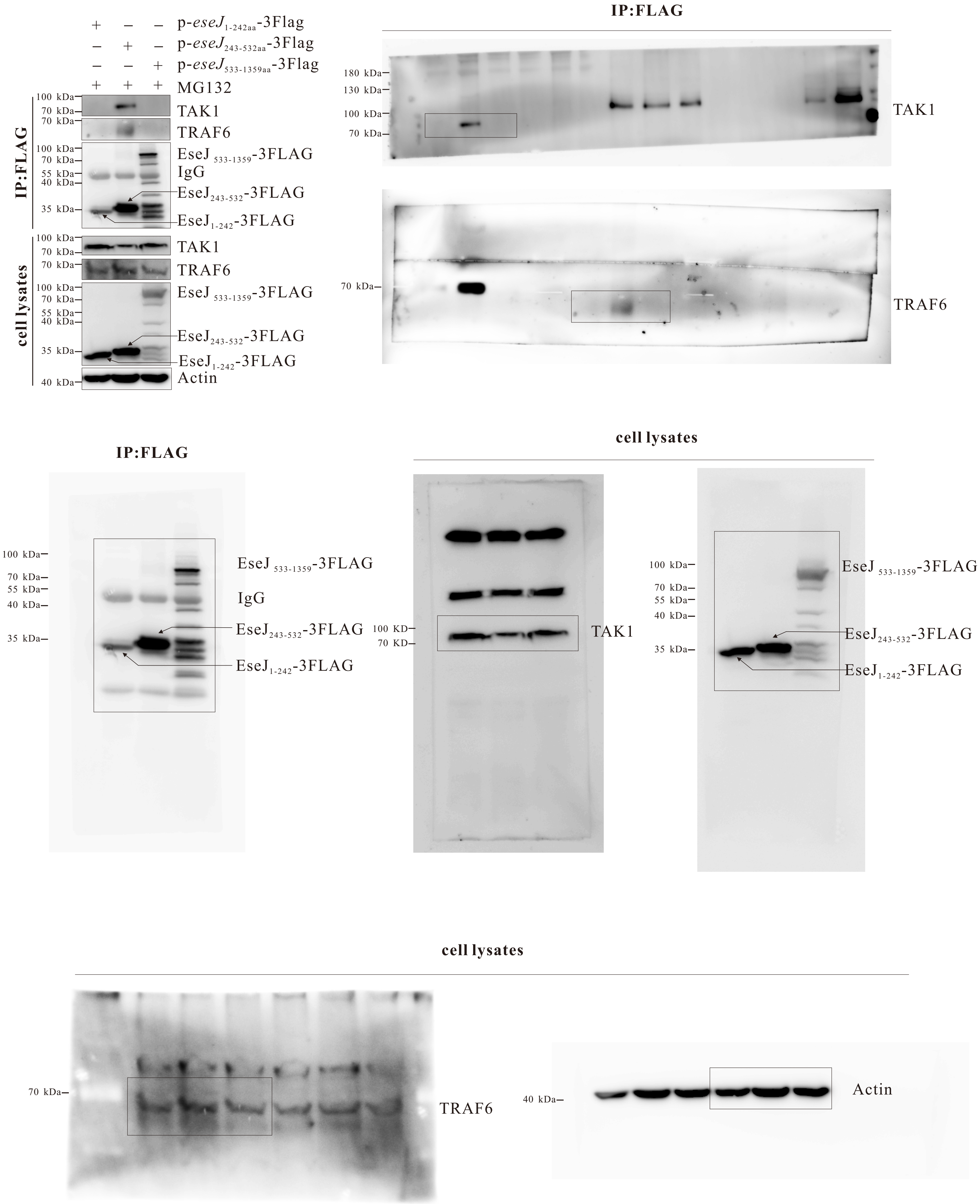

p-eseJ1-242aa-3Flag
p-eseJ243-532aa-3Flag
p-eseJ533-1359aa-3Flag
MG132
IP:FLAG
cell lysates
TAK1
TRAF6
EseJ 533-1359-3FLAG
IgG
EseJ243-532-3FLAG
EseJ1-242-3FLAG
Actin
180 kDa
130 kDa
100 kDa
70 kDa
55 kDa
40 kDa
35 kDa
100 KD
70 KD

Fig 5C

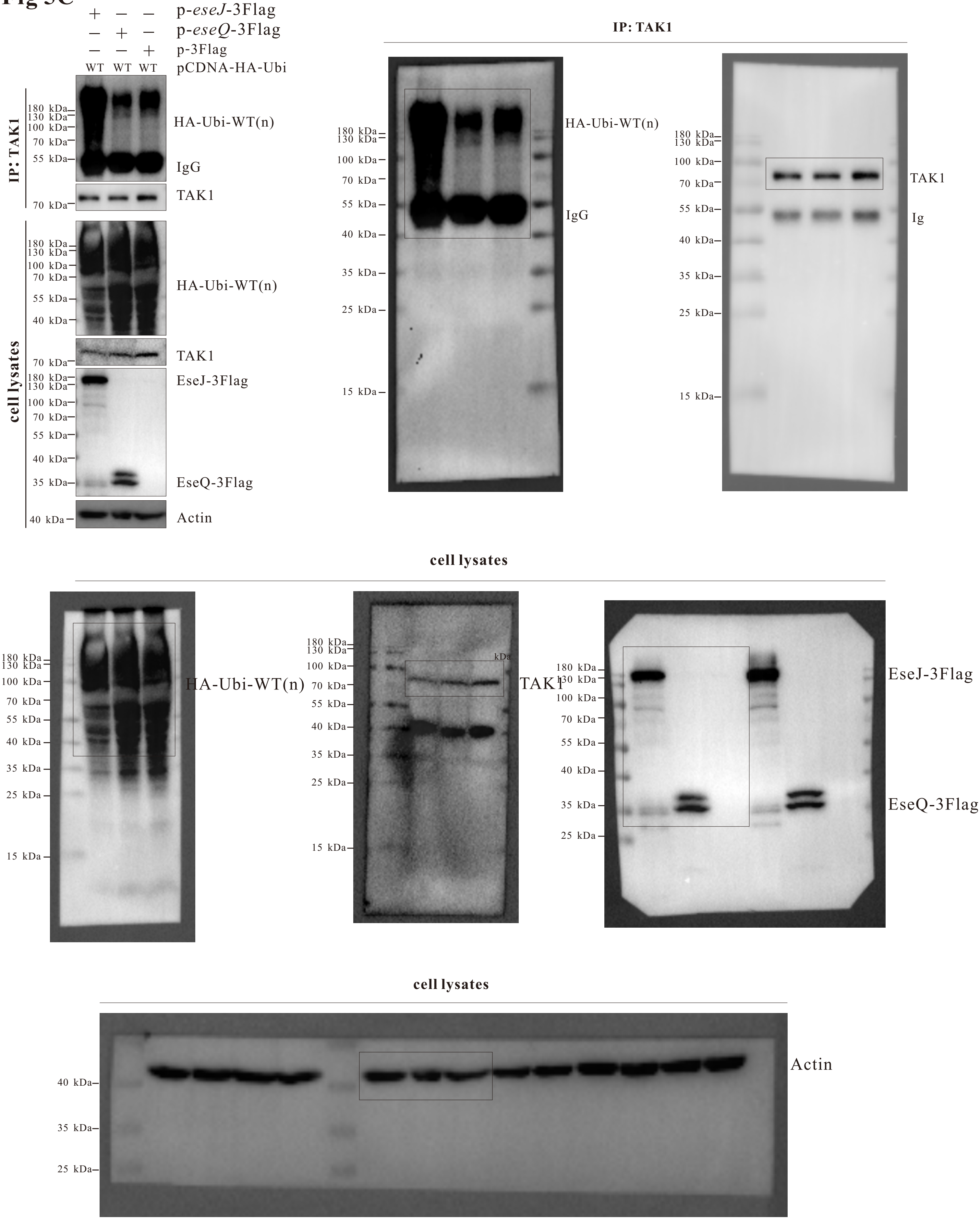

p-eseJ-3Flag
p-eseQ-3Flag
p-3Flag
pCDNA-HA-Ubi
WT WT WT
IP: TAK1
cell lysates
HA-Ubi-WT(n)
IgG
TAK1
Ig
EseJ-3Flag
EseQ-3Flag
Actin
180 kDa
130 kDa
100 kDa
70 kDa
55 kDa
40 kDa
35 kDa
25 kDa
15 kDa

Fig 5D

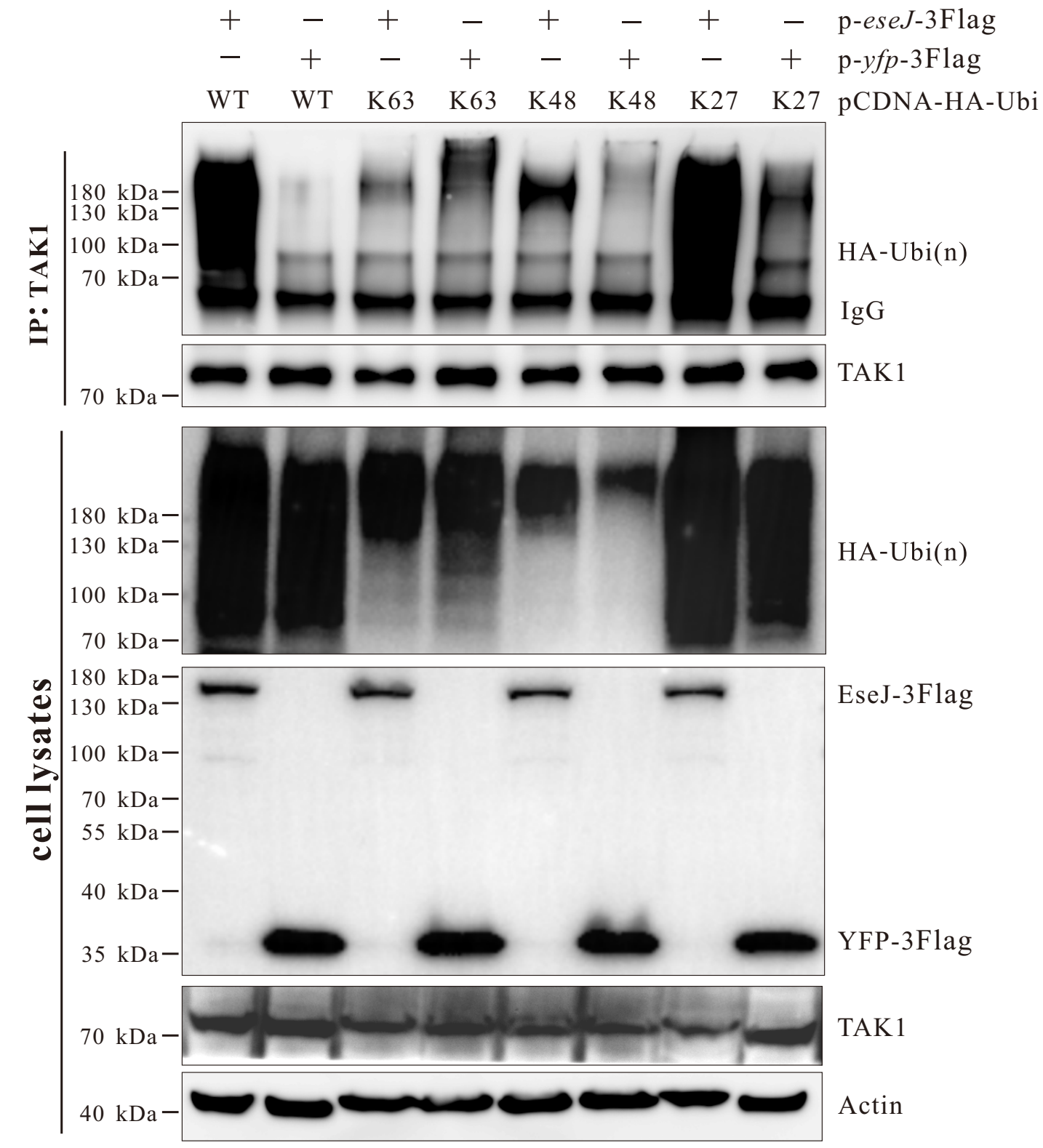

IP: TAK1

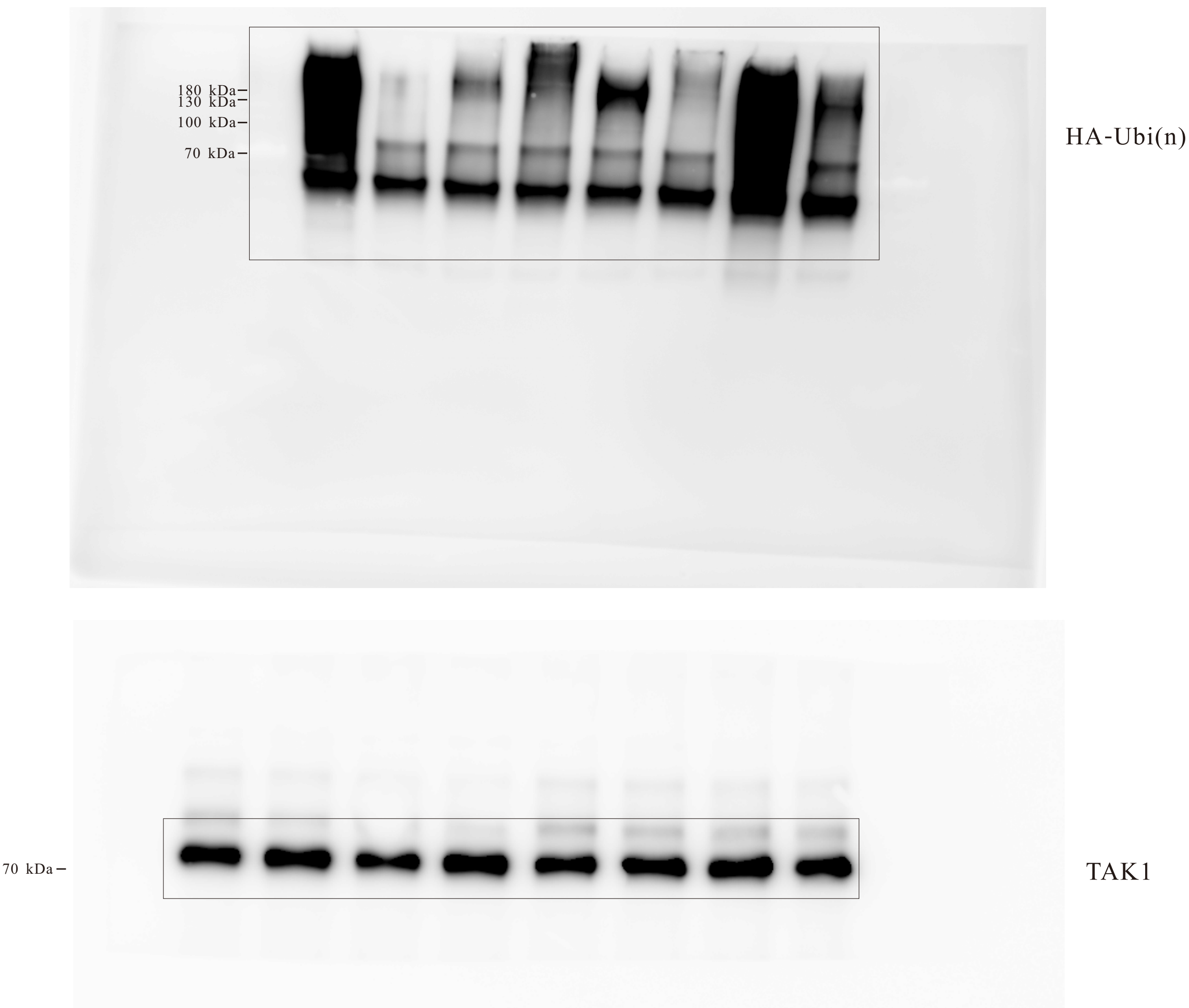

Fig 5D

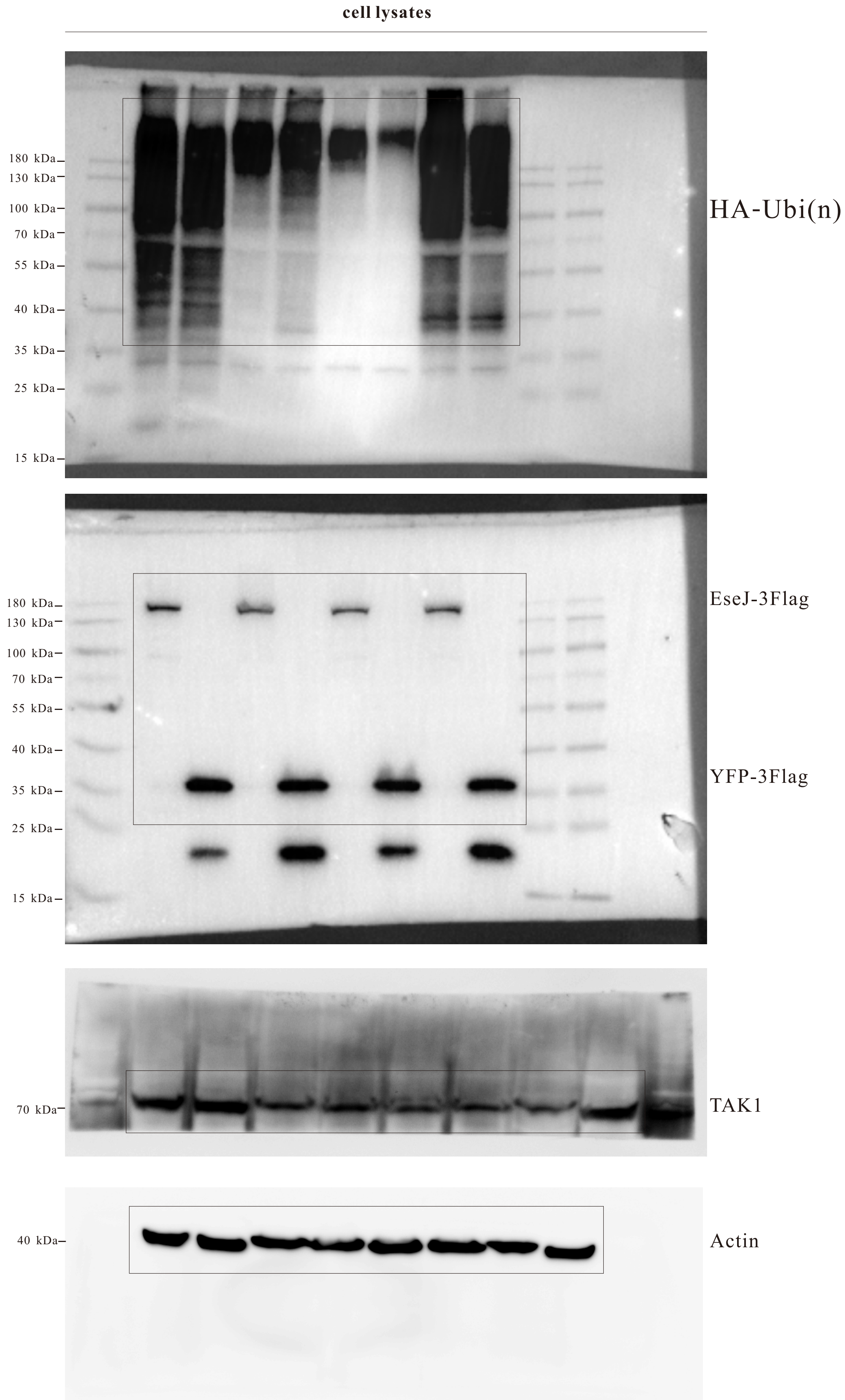
cell lysates
180 kDa
130 kDa
100 kDa
70 kDa
55 kDa
40 kDa
35 kDa
25 kDa
15 kDa
HA-Ubi(n)
EseJ-3Flag
YFP-3Flag
TAK1
Actin

Fig 5E

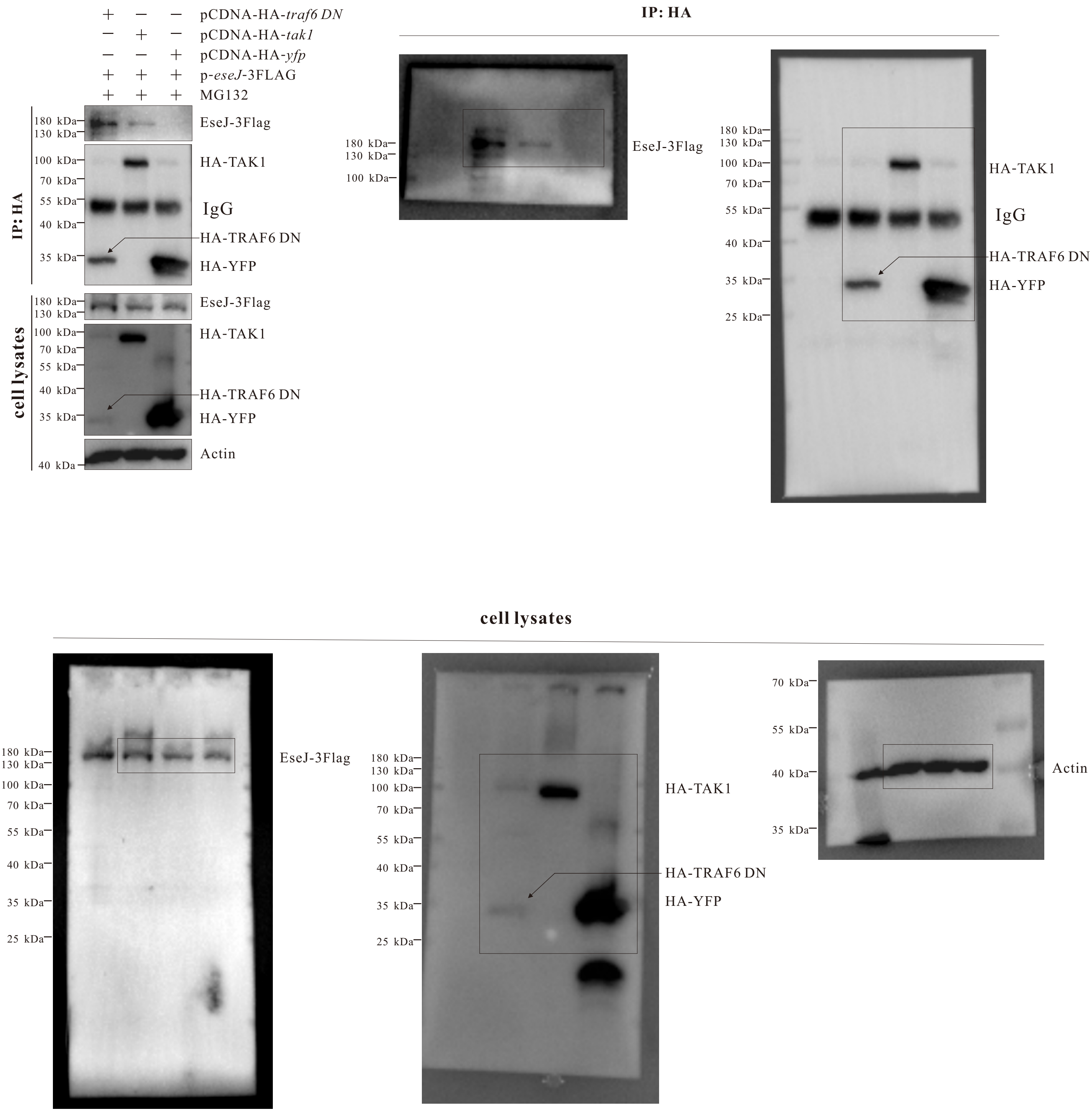

pCDNA-HA-traf6 DN
pCDNA-HA-tak1
pCDNA-HA-yfp
p-eseJ-3FLAG
MG132
IP: HA
cell lysates
EseJ-3Flag
HA-TAK1
IgG
HA-TRAF6 DN
HA-YFP
Actin
180 kDa
130 kDa
100 kDa
70 kDa
55 kDa
40 kDa
35 kDa
25 kDa

Fig 5F

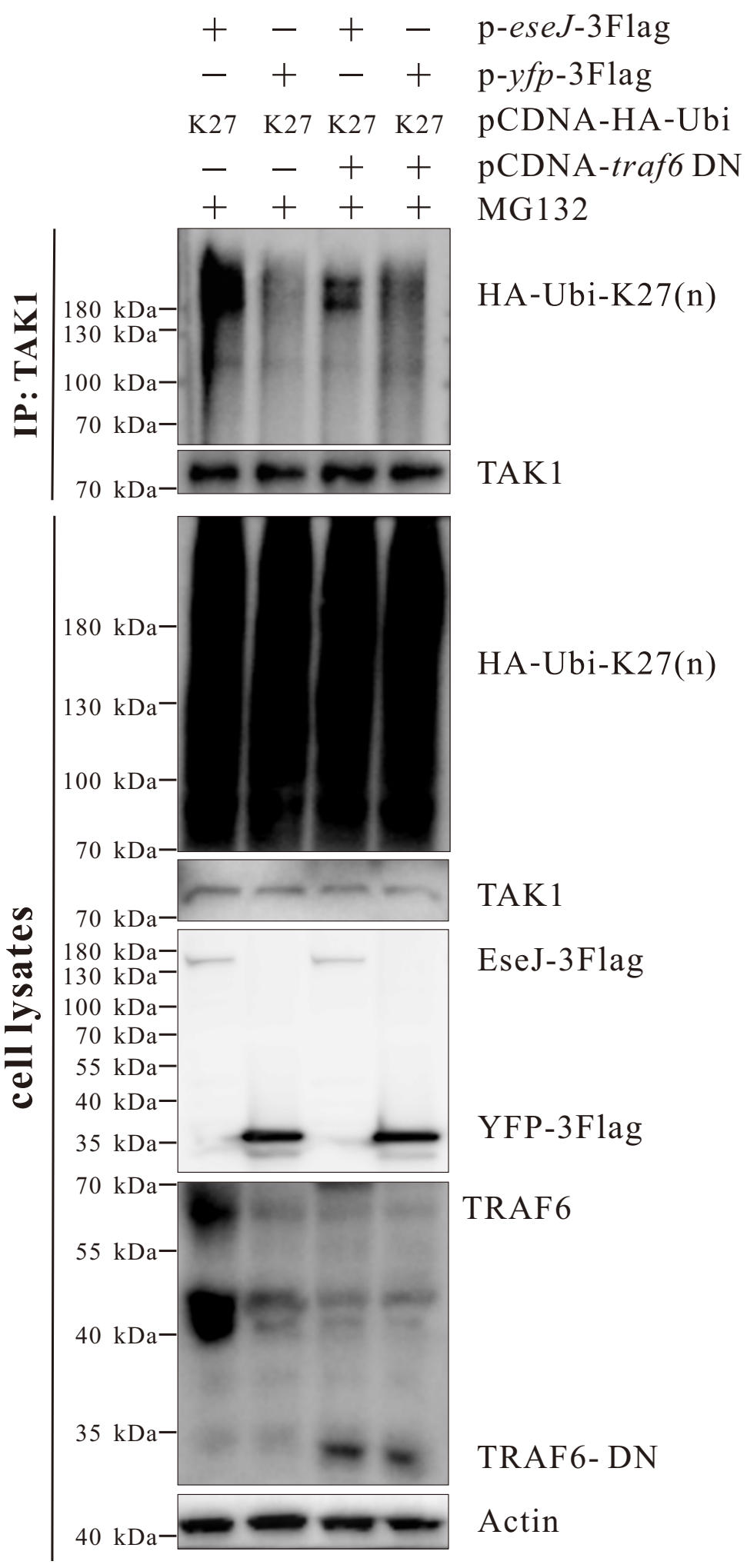

+ − + − p-*eseJ*-3Flag
− + − + p-*yfp*-3Flag
K27 K27 K27 K27 pCDNA-HA-Ubi
− − + + pCDNA-*traf6* DN
+ + + + MG132
IP: TAK1
180 kDa
130 kDa
100 kDa
70 kDa
HA-Ubi-K27(n)
70 kDa
TAK1
cell lysates
180 kDa
130 kDa
100 kDa
70 kDa
HA-Ubi-K27(n)
70 kDa
TAK1
180 kDa
130 kDa
100 kDa
70 kDa
55 kDa
40 kDa
35 kDa
EseJ-3Flag
YFP-3Flag
70 kDa
55 kDa
40 kDa
35 kDa
TRAF6
TRAF6- DN
40 kDa
Actin

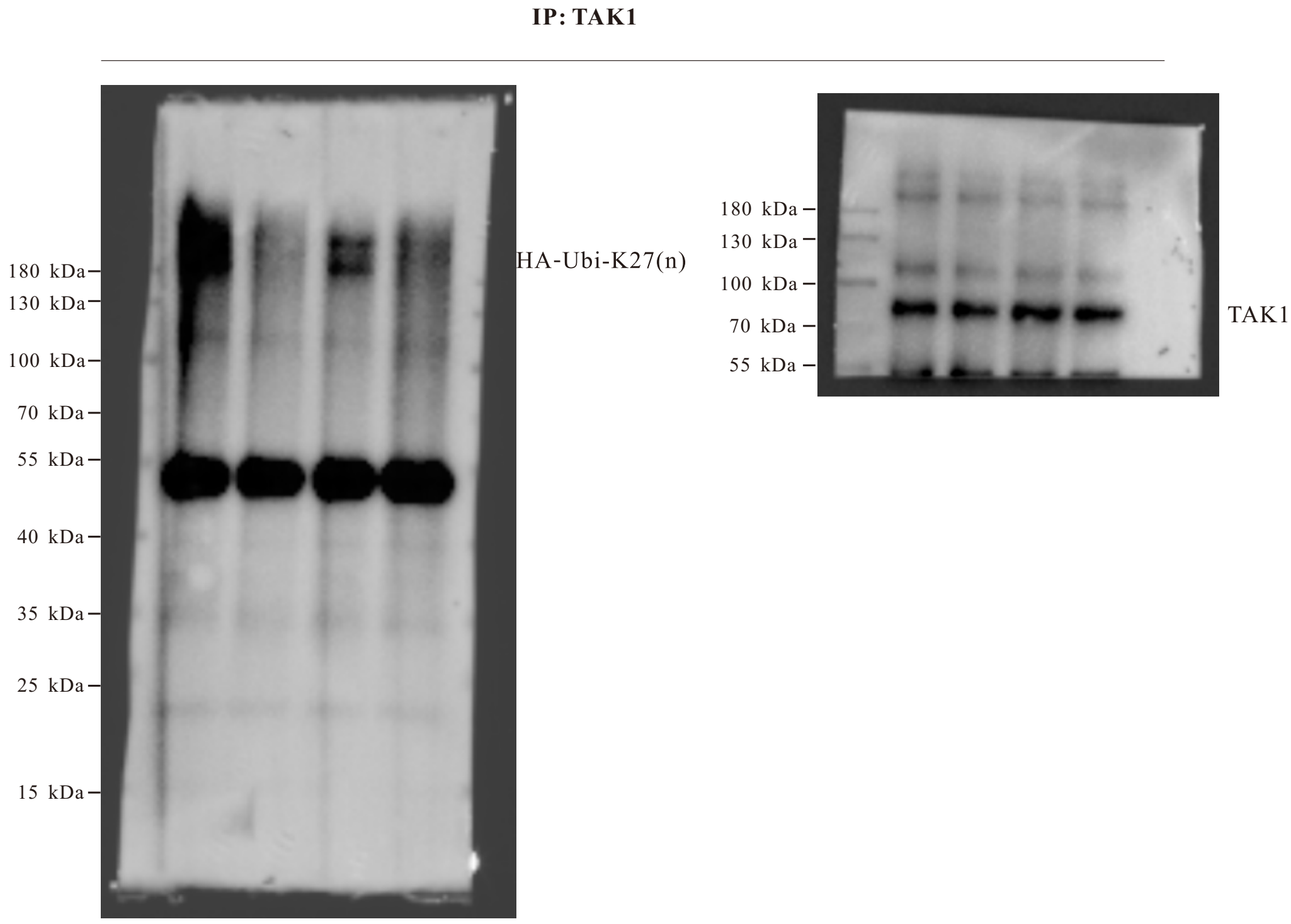

IP: TAK1
180 kDa
130 kDa
100 kDa
70 kDa
55 kDa
40 kDa
35 kDa
25 kDa
15 kDa
HA-Ubi-K27(n)
180 kDa
130 kDa
100 kDa
70 kDa
55 kDa
TAK1

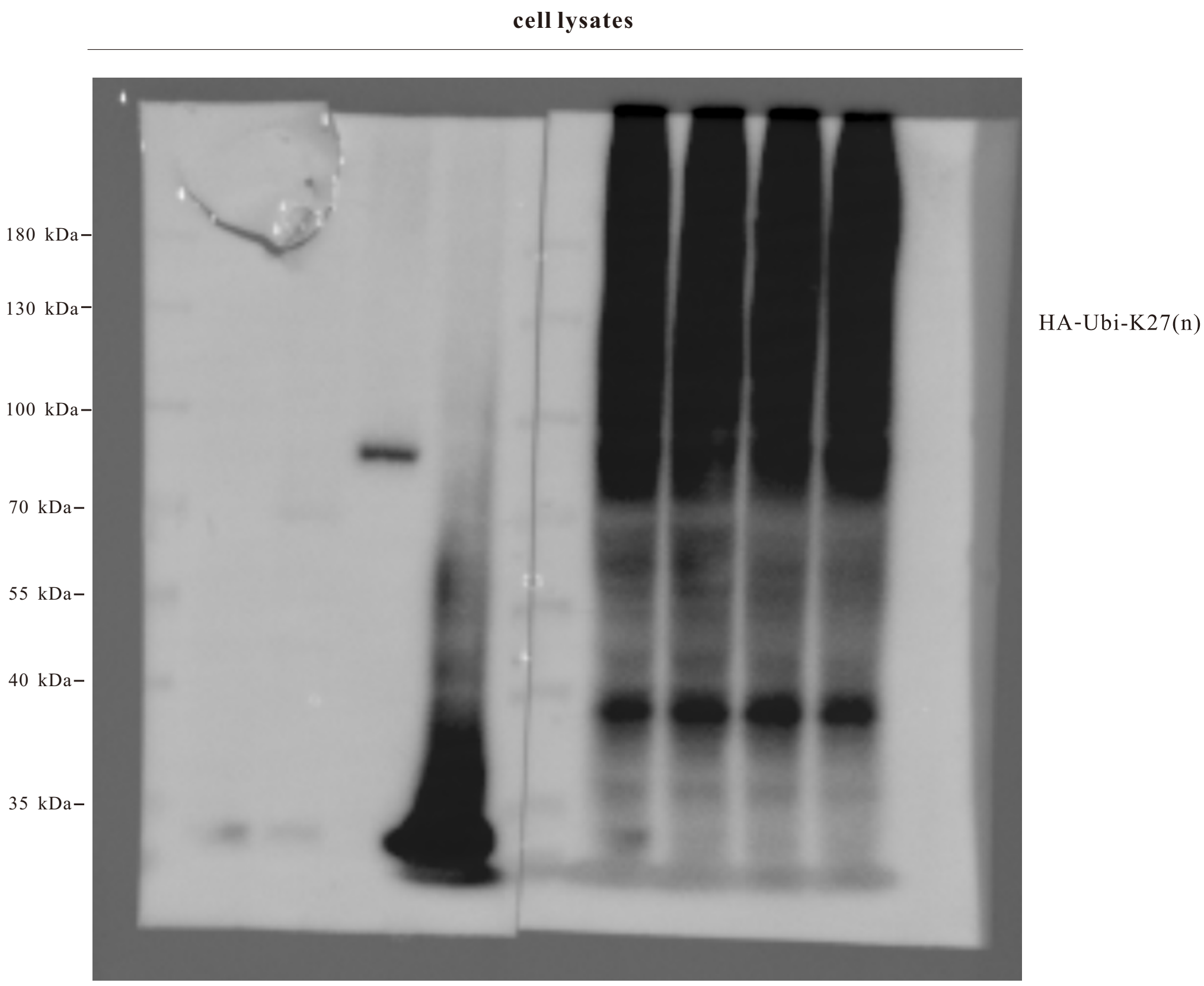

cell lysates
180 kDa
130 kDa
100 kDa
70 kDa
55 kDa
40 kDa
35 kDa
HA-Ubi-K27(n)

Fig 5F

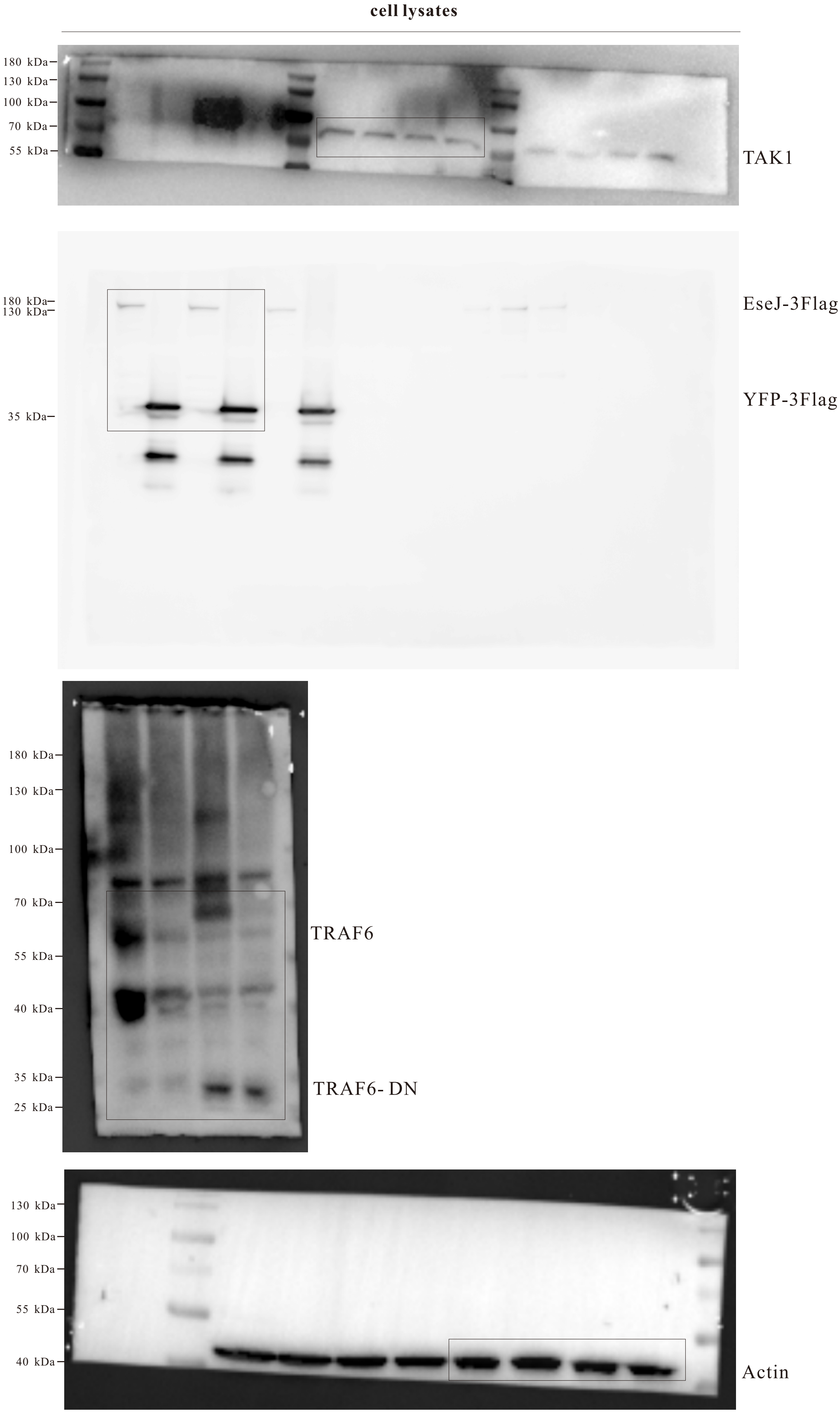
cell lysates
180 kDa
130 kDa
100 kDa
70 kDa
55 kDa
TAK1
180 kDa
130 kDa
35 kDa
EseJ-3Flag
YFP-3Flag
180 kDa
130 kDa
100 kDa
70 kDa
55 kDa
40 kDa
35 kDa
25 kDa
TRAF6
TRAF6- DN
130 kDa
100 kDa
70 kDa
55 kDa
40 kDa
Actin

Fig 6A

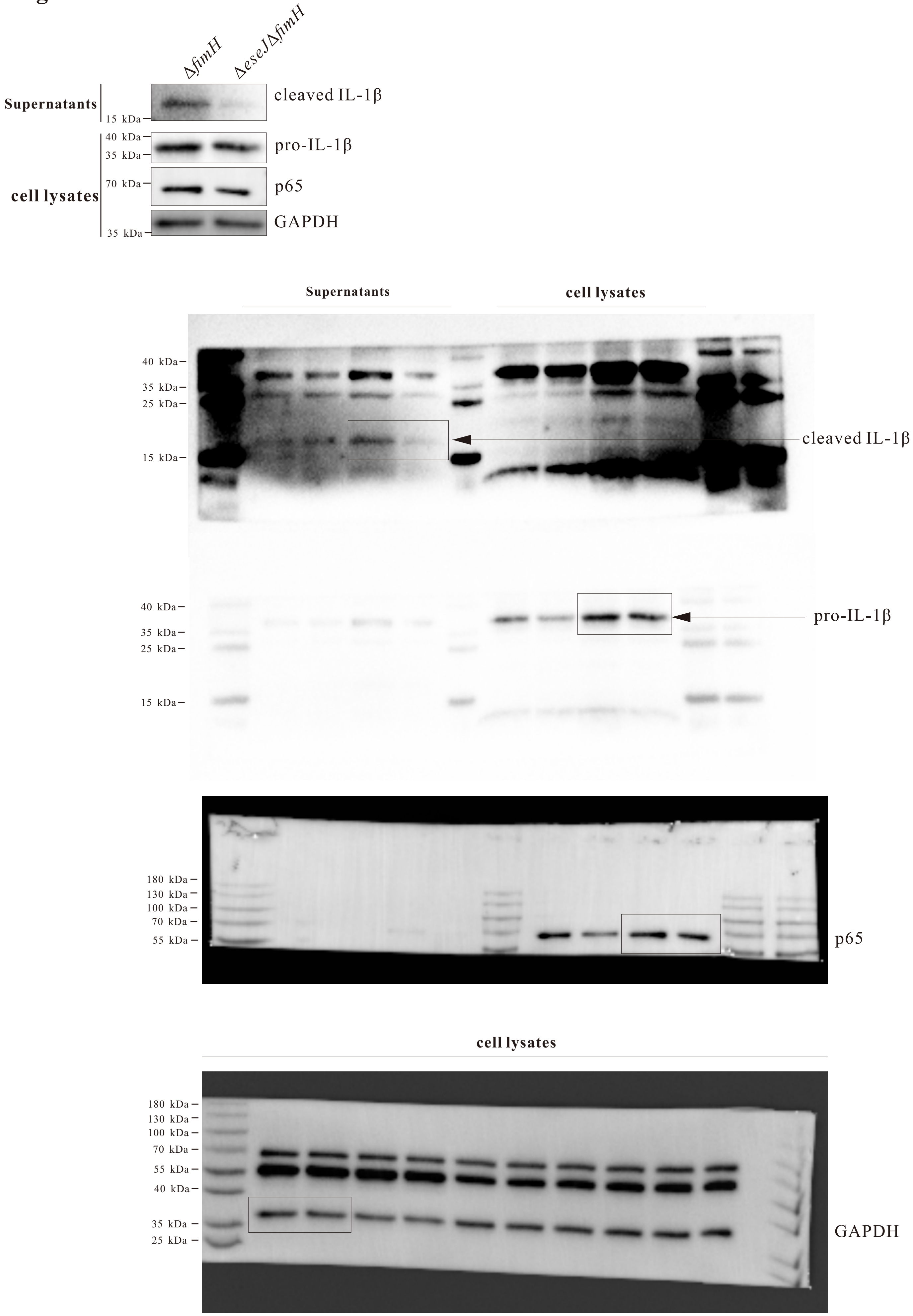
ΔfimH
ΔeseJΔfimH
Supernatants
cell lysates
15 kDa
40 kDa
35 kDa
70 kDa
35 kDa
cleaved IL-1β
pro-IL-1β
p65
GAPDH
Supernatants
cell lysates
40 kDa
35 kDa
25 kDa
15 kDa
cleaved IL-1β
40 kDa
35 kDa
25 kDa
15 kDa
pro-IL-1β
180 kDa
130 kDa
100 kDa
70 kDa
55 kDa
p65
cell lysates
180 kDa
130 kDa
100 kDa
70 kDa
55 kDa
40 kDa
35 kDa
25 kDa
GAPDH
