# Supplementary material for: The Edwardsiella T3SS effector EseJ enhances NF-κB signaling by stabilizing p65 and promoting TAK1 phosphorylation
Source: PLoS Pathog. 2026 Sep 15;22(9):e1014260. doi: 10.1371/journal.ppat.1014260 (PMC13577494; doi:10.1371/journal.ppat.1014260)
Supplement: Supplementary file 2 [file ppat.1014260.s001.docx]

Table S1. Homologies of *E. piscicida* EseJ with proteins in diverse Gram-negative pathogens

| Description of the genes | Accession no | % of identity | % of similarity |
| --- | --- | --- | --- |
| putative T3SS effector Orf 29/30 [*Edwardsiella ictaluri* 93-146] | ABC60092.1 | 80.0 | 85.4 |
| putative T3SS effector  [*Chromobacterium amazonense*] | WP_106076456.1 | 43.0 | 57.6 |
| TTSS effector protein  [*Chromobacterium sinusclupearum*] | WP_103321639.1 | 42.3 | 57.4 |
| hypothetical protein  [*[Chitinimonas sp.](https://www.ncbi.nlm.nih.gov/Taxonomy/Browser/wwwtax.cgi?id=1559339)* [BJB300](https://www.ncbi.nlm.nih.gov/Taxonomy/Browser/wwwtax.cgi?id=1559339)] | [WP_099404680.1](https://www.ncbi.nlm.nih.gov/protein/WP_099404680.1) | 22.4 | 37.6 |
| hypothetical protein  [*Burkholderia dolosa*] | [WP_176077519.1](https://www.ncbi.nlm.nih.gov/protein/WP_176077519.1) | 23.8 | 35.0 |
| hypothetical protein GD429_39280  [*[Burkholderia sp.](https://www.ncbi.nlm.nih.gov/Taxonomy/Browser/wwwtax.cgi?id=2656644)* [BE17](https://www.ncbi.nlm.nih.gov/Taxonomy/Browser/wwwtax.cgi?id=2656644)] | [MPV71674.1](https://www.ncbi.nlm.nih.gov/protein/MPV71674.1) | 8.4 | 12.8 |
| T3SS effector BopA family protein  [*Shewanella baltica*] | WP_310803361.1 | 23.1 | 39.6 |
| T3SS effector BopA family protein  [*Shewanella sp*. NKUCC01_JLK] | WP_219235764.1 | 21.7 | 35.8 |
| T3SS effector BopA family protein  [*Shewanella psychrophila*] | WP_077751501.1 | 23.4 | 38.2 |
| T3SS effector BopA family protein  [*[Vibrio hepatarius](https://www.ncbi.nlm.nih.gov/Taxonomy/Browser/wwwtax.cgi?id=171383)*] | [WP_215989090.1](https://www.ncbi.nlm.nih.gov/protein/WP_215989090.1) | 19.3 | 33.2 |
| T3SS effector BopA family protein  [*Vibrio ostreicida*] | WP_076587940.1 | 20.4 | 35.7 |
| hypothetical protein WCK49_08220  [*Myxococcaceae* bacterium] | MEI6806473.1 | 20.0 | 35.9 |
| hypothetical protein [*Yersinia aleksiciae*] | WP_464059871.1 | 16.8 | 30.4 |
| hypothetical protein [*Yersinia bercovieri*] | WP_222421279.1 | 10.3 | 19.7 |
| hypothetical protein ACRCYL_19395  [*Kluyvera sp*.] | MGL5701340.1 | 11.6 | 20.4 |
